# Enhanced Endophytic Colonization of *Flavobacterium* Promotes Plant Resistance to Fusarium Wilt

**DOI:** 10.1002/advs.77725

**Published:** 2026-09-29

**Authors:** Xianfeng Ye, Changsheng Xu, Shuai Yao, Jiming Shen, Zeyuan Li, Yu Zhang, Fan Wu, Xinjie Li, Yanling Ji, Lei Zhang, Yan Huang, Zhoukun Li, Huayu Qin, Zhongli Cui

**Affiliations:** ^1^ Key Laboratory of Agricultural Environmental Microbiology of Ministry of Agriculture and Rural Affairs Nanjing Agricultural University Nanjing People's Republic of China; ^2^ School of Health Management Zaozhuang University Zaozhuang People's Republic of China

**Keywords:** biofilm, cross‐feeding, *flavobacterium*, microbial consortium, rhizosphere colonization, systemic resistance

## Abstract

Microbial taxa in the soil microbiome with specific ecological functions play crucial roles in plant health. *Flavobacterium* is found closely associated with physiological functions that promote plant growth and enhance disease resistance. While cooperative interactions mediated by metabolite exchange can stabilize microbial consortia, the specific interactions between these consortia and plants remain understudied, particularly regarding their application in plant disease control. Here, we isolate a natural microbial consortium comprising *Achromobacter*, *Flavobacterium*, and *Bacillus* strains from cucumber rhizosphere soil, and identify the *Flavobacterium* strain as a methionine auxotroph. This study reveals the metabolic interactions among strains within a cross‐feeding‐based consortium. The *Achromobacter* and *Bacillus* primarily metabolize carbon and nitrogen sources, respectively, whereas the relevant genes in the *Flavobacterium* are significantly downregulated. The consortium develops significantly enhanced biofilms with a distinct stratified architecture, facilitating root endophytic colonization by *Achromobacter* and *Flavobacterium*. Endophytic *Flavobacterium* induces systemic resistance in plants, thereby enhancing resistance against phytopathogenic fungal infections. Our findings reveal a novel ecological strategy employed by auxotrophic *Flavobacterium* species for both microbial interactions and root endophytic colonization and provide a theoretical foundation for microbial consortium‐based microbial inoculants in plant disease management.

## Graphical abstract

A natural microbial consortium employing a cross‐feeding strategy forms a stratified biofilm that promotes endophytic colonization of auxotrophic *Flavobacterium*. This enhanced colonization activates plant systemic resistance, effectively suppressing Fusarium wilt. The findings reveal a novel ecological strategy for improving beneficial bacterial establishment and provide a theoretical foundation for consortium‐based biocontrol agents.

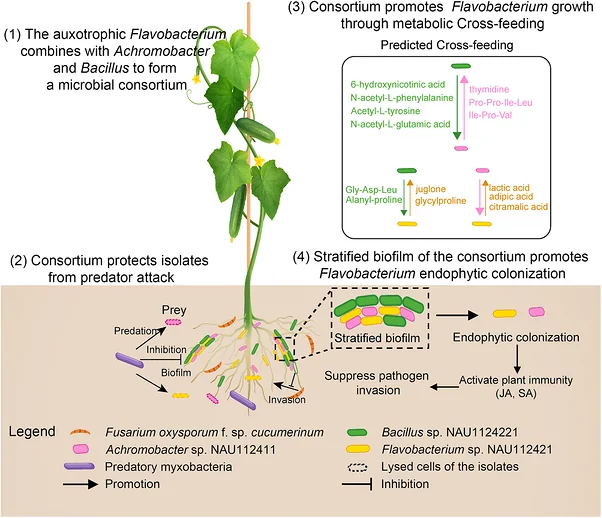

## 1 Introduction

The diversity and structure of microbial communities are critical for ecosystem function on Earth [1, 2]. Specifically, rhizosphere microbes not only participate in biogeochemical processes, but also regulate plant tolerance to biotic and abiotic stresses through plant root colonization. Steering soil microbial communities toward plant‐beneficial configurations is a direction for future soil microbiome research [3, 4, 5]. Although the occurrence of certain microorganisms is governed by stochastic processes, core microbiome members are expected to exhibit substantial stability and reproducibility across diverse spatiotemporal conditions and among distinct healthy plant hosts [6]. Targeted inoculation of these core taxa or keystone modules within a community may constitute an effective strategy for modulating microbial assemblages, offering the dual benefits of reducing agricultural input and increasing crop productivity [7, 8, 9].

*Flavobacterium* is a functional group of microorganisms closely associated with plant physiological processes; these microbes exhibit high relative abundance when colonizing different plant tissues [10, 11]. In the plant rhizosphere and root endospheric compartments, the relative abundance of *Flavobacterium* reached 5.0%–20.2% and 0.53%–20.9%, respectively [11]; however, its relative abundance peaks significantly in the wheat phyllosphere at 41.4% [12]. A higher relative abundance ensures the execution of ecological functions, such as enhancing nutrient absorption by plants through increased material cycling. During the early stages of plant growth, *Flavobacterium* participates in approximately 70% of the carbon cycling in the rhizosphere [10, 13] and can promote plant growth through organic phosphorus mineralization [14]. *Flavobacterium* is the only member of the phylum Bacteroidetes capable of gliding on solid surfaces [10]. Pectin from plant cell walls not only serves as a carbon source for *Flavobacterium* growth but also promotes their surface colonization on plant surfaces [15].

In the complex rhizosphere and endospheric microbiomes, *Flavobacterium* is recognized as a core taxon that plays a pivotal role in plant disease control [16, 17]. Carrión et al. revealed that sugar beets suppresses fungal infections by enriching for *Chitinophaga* and *Flavobacterium* within its roots [16]. Reconstruction of the disease‐suppressive consortium and gene mutation indicated that an unidentified NRPS‐PKS gene cluster derived from *Flavobacterium* plays a key role in inhibiting fungal invasion [16]. The *Flavobacterium* sp. strain TRM1, isolated from the rhizosphere of disease‐resistant tomato, effectively suppressed bacterial wilt caused by *Ralstonia solanacearum* in susceptible tomato cultivars [17]. Although researchers recognize the important role of *Flavobacterium* in plant health, studies exploring its plant rhizosphere colonization and its interactions with both other soil microbes and host plants remain limited.

Predatory myxobacteria are recognized as a novel type of biocontrol microorganisms capable of exhibiting broad‐spectrum predatory activity against various microbes, including both bacteria and fungi, by secreting predatory factors and fungal cell wall‐degrading enzymes [18, 19, 20, 21]. Our previous studies showed that the myxobacterium *Corallococcus* sp. EGB directly affects the phytopathogenic fungus *Fusarium oxysporum* f. sp. *cucumerinum* (FOC) and suppresses the occurrence of plant diseases by regulating soil microbial communities [22]. The relative abundance of *Flavobacterium* in cucumber rhizosphere soil significantly increased after EGB strain treatment [22], although it could be efficiently predated. To clarify the role of *Flavobacterium* in the myxobacterial predation‐driven community, culturable bacteria were isolated from the rhizosphere soil. During this process, a consortium of AFB (Ac/Fl/Ba) composed of *Achromobacter* sp. NAU112411 (Ac), *Flavobacterium* sp. NAU112421 (Fl), and *Bacillus* sp. NAU1124221 (Ba) was obtained (Figure S1). We hypothesize that the consortium not only attenuates the predation of myxobacteria on *Flavobacterium*, but also enhances plant resistance to Fusarium wilt by promoting rhizosphere colonization of bacterial isolates. The results revealed that the cross‐feeding‐driven consortium not only promoted the growth of methionine auxotrophic *Flavobacterium* but also facilitated its endophytic root colonization through the formation of a stratified biofilm structure; thereby inducing plant immunity and reducing disease incidence. This study deciphered the interactions among consortium members and provided a novel strategy for improving endophytic colonization by *Flavobacterium*.

## 2 Materials and Methods

### 2.1 Isolation and Cultivation of Strains

In this study, three isolates were separated from the AFB consortium using the streak plate method [23] and were phylogenetically identified based on the 16S rRNA gene using universal primers (27F and 1492R). All isolated strains were cultured using Reasoner's 2A (R2A) medium [24] and stored at −80°C in 15% (v/v) glycerol (Sigma‐Aldrich, Japan) as a cryoprotectant. The detailed identification protocol for the amino acid auxotrophs of *Flavobacterium* is provided in the Supporting Information and Methods (lines 25–35). *Corallococcus* sp. EGB (CCTCC M2012528) and *Escherichia coli* DH5α (ThermoFisher Scientific, USA) were cultured in LBS and LB medium, respectively, as previous described [22].

Fungal phytopathogen including FOC (ACCC 30220) and *F. oxysporum* f. sp. *lycopersici* (FOL) strain 4287 were cultured in 250‐mL flasks containing 100 mL of potato dextrose broth (PDB) medium [25] at 28°C for 3 d, and the conidia were obtained by removing the mycelia with three layers of sterile microscope lens paper. The conidial concentration was measured using a hemocytometer (XB‐K‐25; Qiujing, China). Interactions between bacterial isolates and strain EGB and FOC were analyzed using lawn predation [22] and colony confrontation assays [21], respectively. Detailed protocols are described in Supporting Information and Methods (lines 36–71).

### 2.2 Biofilm Formation, Quantification, and Microscopic Observation

Three bacterial isolates, *Bacillus velezensis* SQR9 and its derivative mutants, were cultured overnight in Luria–Bertani (LB) medium [22], collected by centrifugation at 8 000 × g for 3 min, and then resuspended in LP liquid medium (a 1:1 mixture of LB and PDB media) to a concentration of 1.0 × 10^8^ CFU/mL. The consortia were obtained by mixing equal volumes of two to three bacterial isolates. Then, 40 µL of bacterial isolates or consortia were inoculated into 96‐well plates containing 160 µL of LP liquid medium each well. The 96‐well plates were incubated at 30°C for 24 h. Sterile LP medium was used as a negative control. The biofilm content of each well was quantified using the crystal violet staining method as previously reported [26, 27, 28].

Additionally, biofilms formed by co‐cultured isolates in 6‐well plates were used to analyze both the bacterial population within the biofilms and the biofilm structure. Total DNA and RNA from the biofilms were extracted using a Bacterial DNA Kit and Bacterial RNA Kit (Omega Bio‐tek, Inc., Norcross, USA), respectively. Scanning electron microscopy (SEM) (SU8010 SEM, Hitachi, Japan) and confocal laser scanning microscopy (CLSM, Leica TCSSP3, Germany) were used to observe the biofilm structures formed by the AFB consortium in LP medium and on cucumber root surfaces. Detailed protocols for the aforementioned experiments are presented in Supporting Information and Methods (lines 72–165).

### 2.3 Greenhouse Experiment

The grouping and objectives of the greenhouse pot experiments are listed in Table S1. The bacterial isolates were cultured in R2A liquid medium at 30°C in a shaker at 180 rpm for 24 h. Then, the cells were harvested by centrifugation, washed twice with sterile ddH_2_O to remove residual medium, and finally resuspended in sterile ddH_2_O to a concentration of approximately 3.0 × 10^9^ CFU/mL. Cucumber seeds were pretreated and germinated as previously reported [22], and transferred to a hydroponic system containing 1/2 MS liquid nutrient medium for further cultivation. Each treatment consisted of five pots (φ 22 cm × 15 cm), with three cucumber plants per pot. Each plant was labeled, and one cucumber plant was randomly selected from each pot to form a repeat. As a result, 15 plants were divided into three replicates, with five plants per replicate. Each pot contained 2.5 kg soil and 0.5 kg organic matter, and conidia of FOC were evenly inoculated into the soil at a final concentration of 10^5^ spores/g soil. The cucumber seedlings with two true leaves were transplanted, and then 3.3 mL of the bacterial isolate suspension was added to the roots of each cucumber seedlings. The final concentration of each isolate in soil was approximately 1.0 × 10^7^ CFU/g soil. The pots were marked and randomly placed in a greenhouse to minimize the impact of environmental factors such as temperature and light on plant growth. The temperature and relative humidity of the greenhouse were maintained in the range of 30°C–35°C and 65.0%–80.0%, respectively. Cucumber symptoms were classified into four levels as previously described [22] and were observed and recorded every two weeks. The disease index of each treatment was calculated as following equation: disease index = [∑(rating × number of plants rated)/(total number of plants × highest rating)] × 100%. At the end of the experiment, soil sampling and DNA extraction were performed as previously described [22]; detailed protocols are provided in Supporting Information and Methods (Lines 166–178). Subsequently, the abundances of bacterial isolates in the bulk soil, rhizosphere soil, and roots were quantified using quantitative PCR (qPCR).

### 2.4 Detection of Transcription Levels of Plant Disease Resistance‐Related Genes

To analyze whether the treatment of bacterial isolates could enhance plant immunity, the transcription levels of disease resistance‐related genes in the root cells of plants from each treatment were measured by destructive sampling on days 1, 3, 7, and 15 after treatment with bacterial isolates. Groups were established in the same manner as in the first patch of the pot experiment. Three cucumber plants were randomly selected from the three pots per group. Cucumber roots were washed with ddH_2_O and dried with absorbent paper. Then, total RNA of samples was extracted by the Spectrum Plant Total RNA Kit (STRN50, Sigma‐Aldrich, USA) and reverse‐transcribed into cDNA using the PrimeScript RT reagent Kit (RR037A, Takara, Dalian, China), respectively, following the instructions. Finally, the transcription levels of plant disease resistance‐related genes were quantified using the 2^−ΔΔCT^ method [22, 29] on a QuantStudio 6 Flex PCR system (Applied Biosystems, USA) with the ChamQ Universal SYBR qPCR Master Mix kit (Vazyme, Nanjing, China). The primers used to detect plant disease resistance‐related genes are listed in Table S2.

### 2.5 Quantification of Bacterial Isolates and FOC Pathogen

The cell numbers of each isolate in the biofilm, liquid medium, soil, and root endosphere samples were determined by qPCR using the QuantStudio 6 Flex PCR system. Specific qPCR primer pairs were designed using Premier 5 (Version 5.0), and are presented in Table S3. Standard curves were obtained using plasmids containing the corresponding fragments [30]. The reagents and protocols for qPCR were as described previously [22].

### 2.6 Transcriptomic Analysis

Total RNA obtained from the biofilms was sent to Shanghai Majorbio Biopharm Technology Co., Ltd (Shanghai, China) for sequencing. Pair‐end read libraries were constructed using the TruSeq Stranded Total RNA Library Prep Kit, and sequencing was performed on an Illumina HiSeq platform. Bowtie software (version 2–2.2.3) was used for quality control of the raw reads and mapping them to the reference genome [31]. Gene functions were predicted by mapping to the KEGG Orthology (KO) database using EggNOG‐mapper (version 2.0) software [32]. The DESeq2 R package was used to analyze differential gene expression [33]. The false discovery rate (FDR) was controlled using Benjamini and Hochberg's method based on the calculated *p* values of genes [30]. Genes were considered differentially expressed if the log2 fold change (LFC) was greater than 2 and FDR was less than 0.05. The primers used to validate the transcriptome sequencing accuracy are presented in Table S4.

### 2.7 Metabolome Analysis Based on LC‐MS System

Three isolates were inoculated into 250‐mL flasks containing 100 mL of M9 glucose medium at a 1.0% inoculum concentration. Then the flasks were incubated in a shaker with 30°C, 180 rpm. The supernatant of each bacterial isolate was collected by centrifugation after all the glucose in the medium was consumed. The concentration of glucose in the medium was measured using Glucose (HK) Assay Kit (Sigma‐Aldrich, USA). The supernatant of each bacterial isolate was filter‐sterilized and inoculated with two other bacterial isolates at 1.0% inoculum (OD_600_ = 1.0). The flasks were incubated under the same conditions for an additional 4 d. Finally, the growth of the bacterial isolates was evaluated by measuring the values of OD_600_ and cell counting.

Two rounds of extracellular metabolic supernatants were collected and sent to Shanghai Majorbio Biopharm Technology Co., Ltd for metabolomics analysis using a UHPLC‐MS/MS system. Sample pretreatment and compound identification were conducted as described previously [30]. Based on the metabolomics results, M9 medium supplemented with a sole carbon source was used to identify the key metabolites in metabolic syntrophy. The concentration of each carbon source is listed in Table S5. Detailed experimental protocols for sample pretreatment for metabolomic analysis and for analyzing the effects of compounds on the growth of isolates are provided in the Supporting Information and Methods (lines 179–212).

### 2.8 Verification of Plant Immune Induction

To determine whether the bacterial isolates induced systemic immunity correlates with plant defense against pathogenic fungal infection, wild‐type and key immunomodulatory gene mutants of *Arabidopsis thaliana* were treated with microflora and subsequently with the FOL pathogen [34] to analyze the severity of infection in plants. The Columbia (Col‐0) ecotype of *Arabidopsis thaliana* and its mutant lines, including *ΔPAL* (GeneID: AT2G37040; SALK number: SALK_000357C) and *ΔLOX* mutants (GeneID: AT1G17420; SALK number: SALK_147830C), were purchased from AraShare. The primers used to analyze the genotypes of the lines using T‐DNA PCR are shown in Table S6.

*Arabidopsis* seeds were soaked in 5% sodium hypochlorite for 5 min and rinsed with sterile ddH_2_O. They were then treated with 70% ethanol for 10 min, followed by three washes with sterile ddH_2_O. Finally, the seeds were incubated in a 0.05% tween−80 solution and kept at 4°C for 2–3 d. Seeds were then transferred and evenly arranged on 1/2 MS solid medium plates using a pipette in a sterile environment. The plates were vertically placed in an incubator at 28°C for 7–10 d. Finally, the *Arabidopsis* seedlings were transferred into soil with 50% peat and 50% vermiculite. The conidia of the FOL pathogen were uniformly sprayed into the soil at a concentration of 10^5^ spores/g soil before the seedlings were transplanted. Aliquots (0.5 mL) of each bacterial isolate suspension (OD_600_ = 2.0) were added to seedling roots. FOL and the consortium were inoculated on the same day as *Arabidopsis* seedling transplantation. Lastly, *Arabidopsis* seedlings were cultured in chambers under the same aforementioned growth conditions. Disease symptoms were observed and recorded every two weeks.

### 2.9 Statistical Analysis

All analyses of microbial interactions, including predation and antagonism experiments, biofilm quantification, compound utilization by bacterial isolation, and analysis of the transcriptional levels of plant immunity‐related genes, were performed with three biological replicates per treatment group. For the quantitative detection of bacterial isolations from soil and root tissues, each treatment group contained three biological replicates (each consisting of three technical replicates). For measurements of biofilm surface area and bacterial isolates’ abundance within the biofilms and the underlying liquid, each treatment comprised six replicates. All results are expressed as the mean ± standard deviation (SD). One‐way analysis of variance (ANOVA) followed by Tukey's multiple comparison test was used to analyze significant differences between treatments. Tukey's Honestly Significant Difference (HSD) test was conducted as a two‐tailed test with 95% confidence interval, therefore, a *p*‐value ≤ 0.05 indicated statistically significant difference between treatment. For transcriptomic analysis, low‐quality reads and adapter sequences were removed using fastp before downstream analysis. Gene expression levels were quantified as fragments per kilobase of transcript per million mapped reads (FPKM), while raw read counts were used for differential expression analysis with DESeq2 package. For metabolomic analysis, raw peak intensities were processed following quality‐control procedures and normalized to the corresponding internal standards to minimize technical variation and improve comparability across samples. For both transcriptomic and metabolomic analyses, *p*‐value for differentially expressed genes and metabolites were corrected using the Benjamini–Hochberg method to control the false discovery rate (FDR). All statistical analyses were performed using SPSS software (version 26.0; IBM Corp., USA). Figures were generated using ggplot2 in the R package or GraphPad prism 9.0. R packages were executed using the R software (version 4.1.0).

## 3 Results

### 3.1 Consortium AFB Significantly Enhances the Ability of *Flavobacterium* to Cope with Biotic Stress

Previous research has shown that the relative abundances of *Flavobacterium* and *Bacillus* were significantly increased in EGB‐treated rhizosphere soil [22]. However, the reason for the significant increase in *Flavobacterium* abundance remains unknown. We hypothesized that variation in the predatory ability of myxobacteria toward their prey might be responsible for this phenomenon. Therefore, the predation ability of myxobacterium strain EGB toward the bacterial isolates was analyzed by enumerating the surviving prey cells in the presence or absence of the predator using the serial dilution spread‐plate method. As shown in Figure 1A,B, strain EGB exhibited predation capabilities against all three isolates, with the strongest predation effect observed for strain Ac, for which the cell number decreased significantly by approximately 10 000‐fold after EGB treatment. However, compared to single isolates, the residual cell numbers of Ac, Fl, and Ba were significantly increased by 4.4‐, 2.7‐, and 2.5‐fold, respectively, in the EGB‐treated consortium AFB, suggesting that microbial consortia enhance bacterial survival by collectively resisting predation by strain EGB (Figure 1A,B).

**FIGURE 1:**
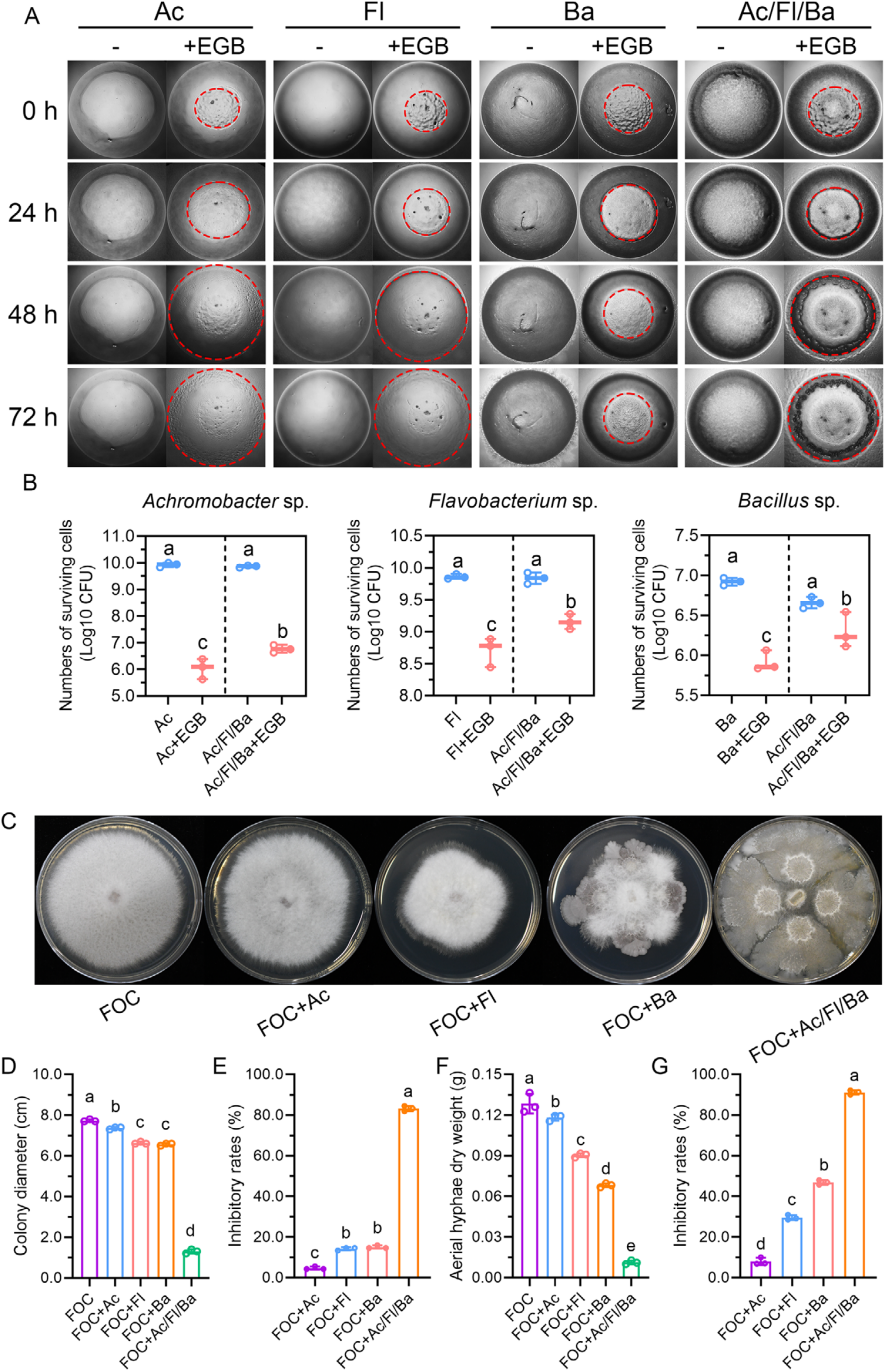
Cooperative interactions among isolates enhance suppression of FOC growth and resistance to predation by myxobacteria EGB. (A) Predation effects of myxobacteria EGB on single isolates and the consortium AFB. The red dashed line marks the diffusion boundary of strain EGB from the colony center to the edge due to predation. (B) The number of residual cells after EGB predation was quantified by the dilution spread‐plate method. (C) Inhibition of FOC growth by single isolates and consortium. (D and F) Colony diameters and the dry weight of aerial mycelium of FOC in different treatment groups, respectively. (E and G) Inhibition rates of different treatments on the colony diameter and aerial mycelium biomass of FOC, respectively. Each treatment was performed in triplicates, and data expressed as mean ± standard deviation (sd). Different lowercase letters above columns indicate significant differences among treatments at *P* ≤ 0.05 based on Tukey's HSD test.

Individual isolates exhibited relatively weak inhibitory effects on the growth of the phytopathogenic fungus, *Fusarium oxysporum* f. sp. *cucumerinum* (FOC). Although strains Fl and Ba showed no significant difference in their inhibition of FOC colony diameter (Figure 1C–E), strain Ba exhibited a markedly stronger inhibitory effect on the aerial mycelial dry weight of FOC, achieving an inhibitory rate of 46.9% compared with 29.6% for strain Fl (Figure 1F,G). In contrast, the AFB consortium (Ac/Fl/Ba) achieved inhibition rates of 83.2% and 92.2% against the colony diameter and aerial mycelial dry weight of FOC, respectively (Figure 1E,G), indicating synergistic interactions among these bacterial isolates. Compared to colonies formed by single isolates, the consortium exhibited significantly enhanced colony expansion capability (Figure 1C and Figure S2). Through stronger spatial competition, the consortium significantly improved the inhibition of the pathogenic fungus FOC (Figure 1C).

### 3.2 Consortium AFB Promotes Auxotrophic *Flavobacterium* Growth Through Metabolic Cross‐Feeding

Both strain Fl and the other *Flavobacterium* isolates originating from cucumber rhizosphere soil were methionine auxotroph (Figure S3). Strain Ba significantly promoted the growth of strains Ac and Fl on M9 glucose solid medium, suggesting the presence of metabolic cross‐feeding among the isolates within the consortium (Figure S4). Metabolic complementation experiments using filtered spent supernatants (with cellular debris removed) from isolates cultured in LP medium were performed to analyze the metabolic promotion between isolates. Except for the spent supernatant from strain Ac, which had no significant growth‐promoting effect on strain Fl, the spent supernatant from all isolates significantly promoted the growth of other strains (Figure 2A,B). Moreover, this growth promotion was more pronounced when the isolates were co‐cultured, suggesting differential resource utilization by the isolates within the consortia (Figure 2A,B).

**FIGURE 2:**
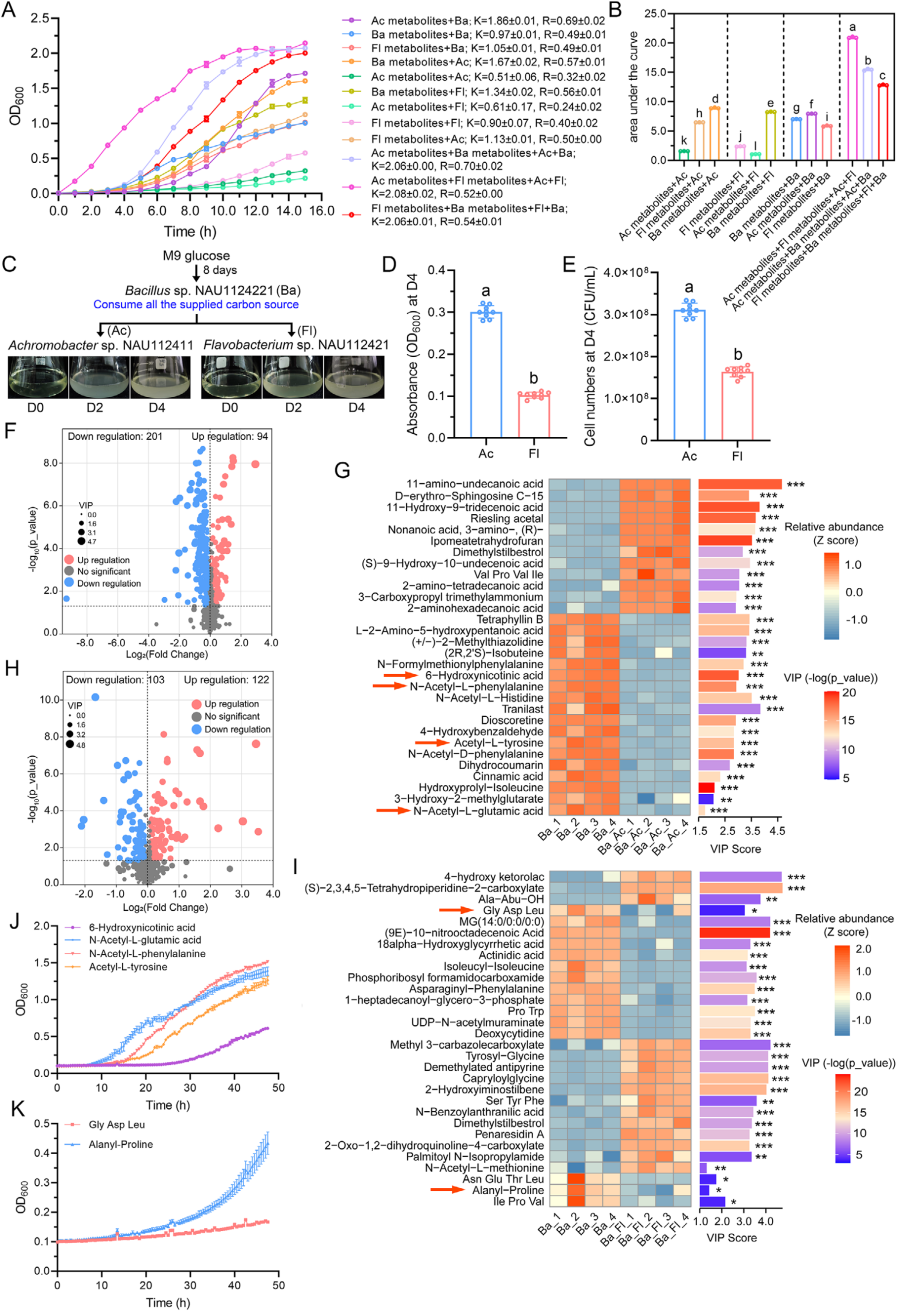
The isolates within the microbial consortium collaborate via metabolic cross‐feeding. (A) Growth curves of three isolates when cultured individually or in coculture using the spent supernatant derived from cultures of each other in LP medium. “K” and “R” represent growth capacity and growth rate, respectively. (B) The area under the growth curve in panel A was calculated using the “growthcurver” package in R software. (C) Schematic of the experiment showing growth stimulation of other isolates by metabolites from a donor isolate. Strain NAU1124221 (Ba) was cultured in M9 glucose medium for 8 d till the glucose could not be detected. The spent supernatant was collected and filtered, then, used as the growth medium for other isolates. “D0, D2 and D4” represent the states of isolates cultured in the spent supernatant for 0, 2 and 4 d, respectively. (D and E) OD_600_ absorbance of cell cultures and cell numbers of isolates in cultures at day 4 in panel (C), respectively. The cell numbers of isolates were determined by the dilution spread‐plate method. The OD_600_ absorbance of the isolates cultured for 4 d in their own spent supernatant was used as control. Since essentially no growth occurred, the OD_600_ absorbance values in control groups were not shown in the bar chart. (F and H) Volcano plots showing differentially abundant metabolites in strain NAU112411 (Ac) and NAU112421 (Fl), respectively, after 4 d of culture in spent supernatant from strain Ba. (G and I) Metabolic profiles of the spent medium experiment. “Ba” indicates the spent supernatant of strain Ba grown on M9 glucose, “Ba_Ac” and “Ba_Fl” indicate the spent medium of strains Ac or Fl grown on cell‐free filtrate of strain Ba. Red arrows indicate substrates utilized by strains Ac and Fl in the spent M9 glucose supernatant from strain Ba. (J and K) Growth curves of strains Ac and Fl, respectively, in the M9 minimal medium supplemented with the tested compounds as the sole carbon source. For cell cultures’ absorbance and isolates’ abundance measurements in panels D and E, each treatment comprised three biological replicates, each with three technical replicates. All other experiments included three replicates per treatment. Values are expressed as mean ± sd. Different letters above columns indicate significant differences among treatments according to Tukey's HSD test at *P* ≤ 0.05.

UPLC‐MS‐based metabolomic analysis was employed to identify the substances involved in cross‐feeding between the isolates. The results showed that all isolates were able to grow and proliferate in the spent supernatants from the other two isolates (Figure 2C–E and Figures S5A–C and S6A–C). Although strain Ba exhibited weaker utilization of the spent supernatants from the other two isolates, Ac and Fl, its cell concentration still reached 9.1 × 10^6^ and 1.3 × 10^7^ CFU/mL, respectively, after 4 d of culture (Figures S5C and S6C). The spent supernatant from the Ba strain significantly promoted the growth of both Ac and Fl (Figure 2D,E). Following cultivation in this supernatant, 201 and 103 metabolites were significantly downregulated in Ac and Fl supernatants, respectively (Figure 2F,H).

The top 30 compounds exhibiting the largest relative abundance changes are displayed in the heatmaps (Figure 2G,I). Commercially available compounds selected from this list were tested for their ability to support the growth of the Ac and Fl strains (Table S5). Among these, 6‐hydroxynicotinic acid, N‐acetyl‐L‐phenylalanine, acetyl‐L‐tyrosine, and N‐acetyl‐L‐glutamic acid secreted by strain Ba supported the growth and proliferation of strain Ac (Figure 2G,J), whereas Gly‐Asp‐Leu and alanyl‐proline were utilized by strain Fl (Figure 2I,K). Correspondingly, thymidine, Pro‐Pro‐Ile‐Leu, and Ile‐Pro‐Val secreted by strain Ac were metabolized by the Ba strain (Figure S5E,H). Unfortunately, many of the compounds significantly depleted in the spent supernatant of strain Ac following utilization by strain Fl were not commercially available; consequently, no specific compounds were identified from this interaction (Figure S5G). Among the metabolic compounds produced by strain Fl, lactic acid, adipic acid, and citramalic acid significantly promoted strain Ac growth (Figure S6D,E,H), whereas juglone and glycylproline were utilized by strain Ba (Figure S6F,G,J). Moreover, compared to Ba alone, the co‐culture of Ba and Fl resulted in significantly reduced contents of methionine, arginine, glutamic acid, valine, proline, and histidine in the supernatant, indicating that Fl may utilize the amino acids secreted by Ba (Figure S7).

### 3.3 *Flavobacterium* Promotes Consortium Biofilm Formation and Benefits from Metabolic Cross‐Feeding

The stronger colony expansion ability of the consortium implied a potentially enhanced capacity for biofilm formation (Figure S2). Although strain Ba exhibited relatively weak biofilm‐forming capabilities, strains Ac and Fl significantly promoted the formation of *Bacillus* biofilms, and the resulting biofilms exhibited distinct morphological characteristics (Figure S8). Consortia containing strain Fl developed biofilms with snowflake‐like clusters (Figure S8). Further investigation revealed that Fl significantly increased the surface area of biofilms by approximately 41.1% by enhancing biofilm wrinkling (Figure 3A,B). The wrinkling appeared as biofilms’ bulges and the formation of “ridges” caused by bacterial clusters (Figure 3A and Figure S9). The increase in biofilm surface area promoted colonization of the isolates within the biofilm (Figure 3C–H). In the biofilm formed by consortium Ac/Fl/Ba, the abundances of strains Ac and Fl reached 1.9 × 10^8^ and 1.5 × 10^7^ copies/cm^2^, respectively (Figure 3F,G). Although co‐culturing of strains Ac and Fl did not result in a discernible biofilm, the growth of these two strains was enhanced (Figure S10).

**FIGURE 3:**
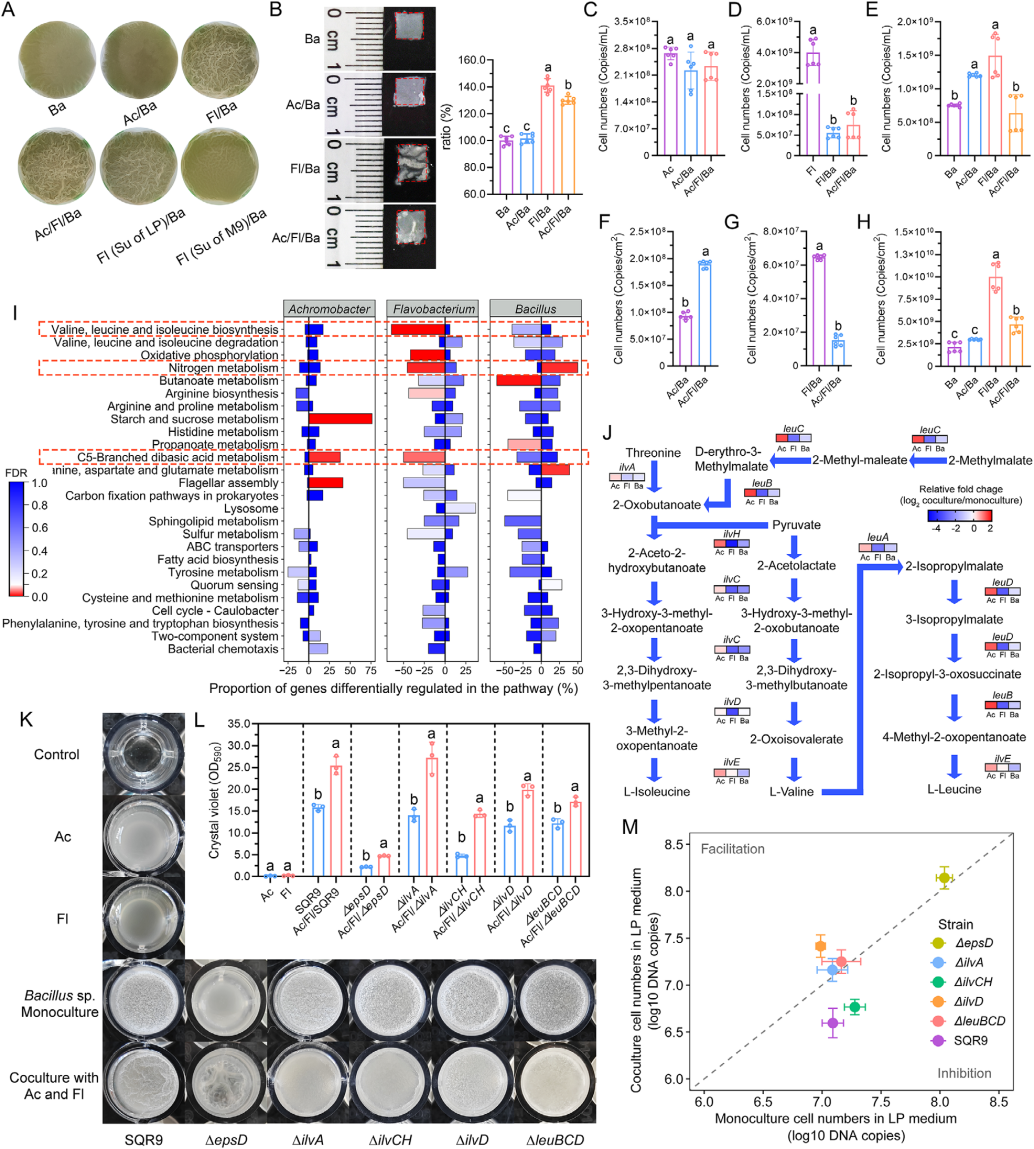
Strains Fl and Ac enhance the consortium biofilm formation by increasing biofilm surface area and upregulating BCAAs synthesis, respectively. (A) Biofilm formed by strain Ba and the consortia in 6‐well plates containing 10 mL/well of LP or M9 medium. “Fl (Su of LP)/Ba and Fl (Su of M9)/Ba” represent treatments where 0.1 g of lyophilized powder from the fermentation supernatant of Fl cultured in LP or M9 medium, respectively, was added to 10 mL of liquid LP medium inoculated with Ba. (B) Biofilm with a side length of 0.5 cm was collected from the 6‐well plate, unfolded, and its surface area was measured. (C–H) The cell numbers of Ac (C and F), Fl (D and G), and Ba (E and H) in the lower liquid layer (C–E) and within the biofilm (F–H) of the 6‐well plate, determined by quantitative PCR. (I) Transcriptional analysis of isolates after 24 h coculture in LP medium. Left and right bars represent proportion of downregulated and upregulated genes in KEGG pathways, respectively. Pathways with LFC > 2 and FDR < 0.05 were considered significantly regulated (highlighted in red). Pathways highlighted in red boxes indicate key pathways that were significantly regulated during coculture. (J) Relative transcriptional levels of genes involved in BCAAs biosynthesis. Color indicates the mean relative fold change of mRNA in each strain during coculture versus monoculture. (K and L) Biofilm formation and quantification of strain SQR9 and its mutants in monoculture or coculture with Ac and Fl in LP medium after 24 h. (M) The cell count in the liquid medium beneath the biofilm using the dilution spread‐plate method in monoculture or coculture with Ac and Fl. For measurements of biofilm surface area (panel B) and bacterial isolates abundance within the biofilm and the underlying liquid (panel C‐H), each treatment included six replicates. All other experiments included three replicates. Values are expressed as mean ± sd. Different letters above columns indicate significant differences among treatments according to Tukey's HSD test at *p* ≤ 0.05.

Transcriptomic analysis of the three isolates under both monoculture and coculture conditions was performed to elucidate the mechanisms underlying consortium formation and enhanced biofilm development. After 24 h of co‐cultivation, 1502, 1441 and 1774 genes were significantly differentially regulated in the Ac, Fl, and Ba strains, respectively. Major transcriptional alterations occurred in genes associated with nitrogen metabolism, butanoate metabolism, amino acid biosynthesis, flagellar assembly, and starch and sucrose metabolism (Figure S11 and Figure 3I). Three metabolic pathways (flagellar assembly, starch and sucrose metabolism, and C5‐branched dibasic acid metabolism) were significantly upregulated in strain Ac during co‐culture (Figure 3I). Within the cells of strain Ba, pathways involved in nitrogen metabolism and amino acid biosynthesis were significantly upregulated (Figure 3I). Interestingly, while genes related to the branched‐chain amino acid (BCAAs) synthesis pathway were downregulated, genes involved in the synthesis of other amino acids (tryptophan, arginine, histidine, alanine, aspartate, glutamate, and methionine) were upregulated in strain Ba (Figure 3J and Figures S12–S15). These results were also validated using qRT‐PCR (Figure S16). However, the reduced production of BCAAs in strain Ba may be compensated for by strain Ac, in which the genes of the valine, leucine, and isoleucine biosynthesis pathways (*ilvB*, *leuB*, *leuC*, and *leuD*) were significantly upregulated by approximately 3.0‐fold (Figure 3J). In contrast, five pathways in the co‐cultured strain Fl were significantly downregulated, and no significantly upregulated pathways were observed (Figure 3I), suggesting that strain Fl may act as a beneficiary within the consortium.

Due to the difficulty in constructing mutant strains of Ba, *B. velezensis* SQR9 and its extracellular polysaccharide (EPS) and BCAAs synthesis mutants (*ΔepsD, ΔilvA, ΔilvCH, ΔilvD*, and *ΔleuBCD*) were selected to validate functional collaboration in the consortium. Similar to strain Ba, coculture with strains Ac and Fl significantly enhanced biofilm formation in SQR9 and the *ΔepsD* mutant (Figure 3K,L). Except significantly increasing biofilms in the mutants, co‐culture also promoted the growth of the *ΔepsD*, *ΔilvA*, *ΔilvD*, and *ΔleuBCD* mutants in LP medium (Figure 3M). All mutants, except for *ΔilvA*, also exhibited a similar increase in biofilm and biomass in nutrient‐limiting MSgg medium (Figure S17). Biofilm formation by SQR9 and its mutants was directly enhanced by 20 ppm BCAAs (Figure S18), suggesting a defined collaborative behavior among the isolates within the consortium.

### 3.4 The Stratified Biofilm Architecture Promotes Efficient Colonization of *Flavobacterium* on Plant Root Surfaces

Greenhouse pot experiments were conducted to evaluate the efficacy of the individual isolates and consortium in suppressing Fusarium wilt. The disease index of the FOC‐treated cucumbers reached 80.0%, whereas the consortium treatment exhibited a disease index of 13.3%, achieving a biocontrol efficacy of 83.4%, which was significantly higher than that of any single isolate (F(9, 20) = 55.94, *p* < 0.0001) (Figure 4A and Figures S19 and S20). The abundance of each isolate in the individual and consortium treatments was significantly higher than in the control and FOC treatment groups (Figure 4B–I). For example, compared to the control, the abundance of Fl in the roots increased 80.5‐, 49.6‐, 217.3‐, and 85.1‐fold in the FOC + Ac/Fl/Ba, FOC + Fl, Ac/Fl/Ba, and Fl treatments, respectively (Figure 4I). Interestingly, the abundance of each isolate in the consortium treatment group was significantly higher than that of the corresponding strain in the single isolate treatment groups (Figure 4B–I). Such as, the abundance of Fl within roots in Ac/Fl/Ba consortium treatment was 3.4 × 10^6^ copies/g root, significantly higher than the 1.3 × 10^6^ copies/g root in the Fl treatment (Figure 4I). Even in the Ba‐only treatment, the abundance of strain Fl in the roots increased by approximately 12.3‐fold compared to the control group (from 1.6 × 10^4^ copies/g root to 1.9 × 10^5^ copies/g root) (Figure 4I). Similar changes were observed in the abundance of Ac within the plant roots (Figure 4H). Moreover, compared with the Ac treatment, the Ba treatment exhibited higher Ac copy numbers in the plant roots (Figure 4H), suggesting that Ba may significantly promote the migration of Ac into the plant roots. Both treatments with individual isolates and the consortium resulted in a significant reduction in FOC abundance in the soil (Figure 4J).

**FIGURE 4:**
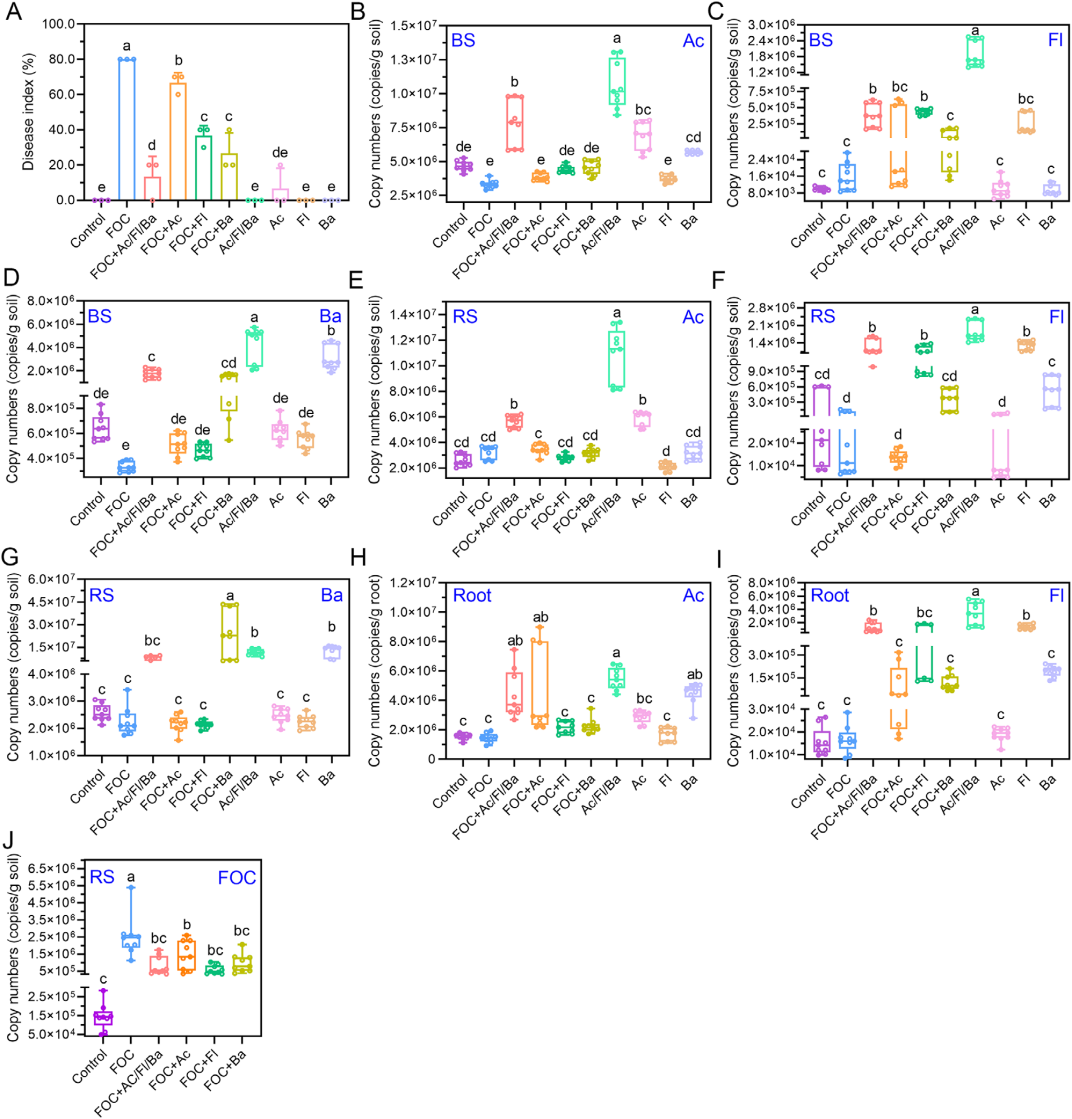
Consortium enhances cucumber resistance to Fusarium wilt by promoting the colonization of isolates in both the rhizosphere and root tissues. (A) Disease index of cucumbers in different treatment groups. (B–I) Copy numbers of Ac (B, E and H), Fl (C, F and I), and Ba (D and G) in bulk soil (BS), rhizosphere soil (RS) and within cucumber roots. (J) Copy numbers of FOC in rhizosphere soil under different treatments. The Copy numbers of all the above isolates and FOC strains were determined by quantitative PCR. The values were expressed as mean ± sd. Disease index was derived from three replicates per treatment in the biocontrol pot experiment. For qPCR, each biological replicate had three technical replicates, and data represent the mean ± sd of nine measurements. Different letters above columns indicate significant differences among treatments at *p* ≤ 0.05 based on Tukey's HSD test.

Subsequently, three fluorescently labeled isolates (Figure S21) were used to analyze the colonization behavior of the Ac/Fl/Ba consortium on the plant root surfaces. Distinct stratified colonization was observed in the consortium of plant roots (Figure 5). Strains Ac and Fl resided within the inner layer of the biofilm formed by *Bacillus*, closer to the root surface cells, whereas strain Ba was located on the outer side (Figure 5A,B). Compared with mono‐colonization, co‐colonization significantly increased the cell numbers of strains Ac, Fl, and Ba on the root surface by 3.7‐, 3.9‐, and 9.4‐fold, respectively (Figure 5C). Transverse section observations of cucumber root tissues revealed that strains Ac and Fl primarily migrated into the roots from lateral root emergence sites and colonized the intercellular spaces of the cortical cells (Figure S22). Additionally, the chemotaxis of the isolates toward cucumber root exudates (with a maximum chemotactic concentration of approximately 10 mg/mL) (Figure S23) and the differential utilization of compounds within these exudates (Figure S24) also facilitated the formation of a consortium and stable colonization of root surfaces.

**FIGURE 5:**
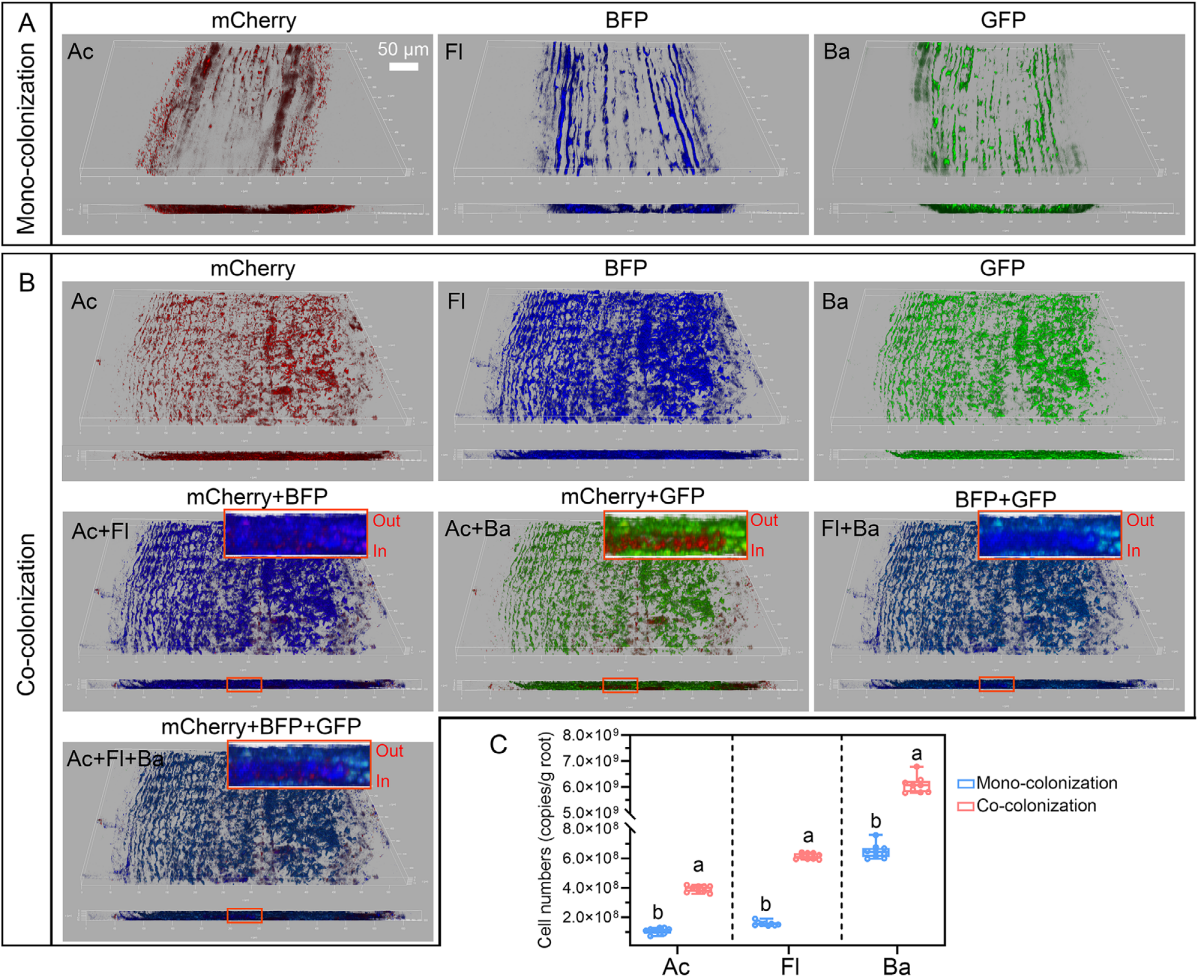
Stratified colonization of isolates from microbial consortium on cucumber root surfaces. Colonization of cucumber root surfaces by microbial isolates in hydroponic systems. Confocal laser scanning microscopy (Leica) images showing mono‐colonization (A) and co‐colonization (B) of the isolates on root surfaces. (C) The number of isolates during mono‐colonization and co‐colonization on cucumber root surfaces determined by quantitative PCR. All treatments were performed with three biological replicates, each with three technical replicates. Values are expressed as mean ± sd of nine measurements. Different letters above columns indicate significant differences among treatments according to Tukey's HSD test at *p* ≤ 0.05.

### 3.5 Enhanced *Flavobacterium* Endosymbiosis Activated Plant Immunity

Previous research revealed that *Flavobacterium* can inhibit the invasion of plant pathogens by inducing systemically acquired resistance in plants [16, 17]. Therefore, the transcription levels of immune‐related genes in plant root tissues treated with both the isolates and the consortium were examined. As shown in Figure 6A–C, compared with normal plants, regardless of whether FOC spores were added to the soil, the transcription levels of the jasmonic acid (JA) pathway genes *LOX*, *AOS2*, and *PBZ1*, were significantly upregulated in response to treatment with Fl alone or in consortium. *PBZ1*, a downstream response protein gene, exhibited delayed transcription compared with *LOX* and *AOS2* (Figure 6A–C). In contrast to the JA pathway, the expression of genes associated with the salicylic acid (SA) pathway, *NPR1* and *PR3*, significantly increased on day 7 of treatment (Figure 6D,E). Additionally, the transcription of *PAL*, a key enzyme in the phenylpropanoid metabolic pathway, was significantly upregulated during the early stages of treatment with strain Fl or the consortium and may potentially contribute to the activation of the SA pathway (Figure 6F). In contrast, transcriptional changes in the *CTR1* gene, which belongs to the ethylene signaling pathway, were minimal across all treatments (Figure 6G). Interestingly, neither strain Ac nor Ba induced significant changes in the transcriptional levels of the aforementioned genes in plant cells (Figure 6B–G). Compared with the groups treated with strain Fl alone, treatment with the consortium significantly enhanced the rhizospheric and endophytic colonization of Fl (Figure 4F,I), along with markedly elevated transcription levels of related genes in both pathways (Figure 6), suggesting that strain Fl plays an important role in the control of Fusarium wilt by the AFB consortium.

**FIGURE 6:**
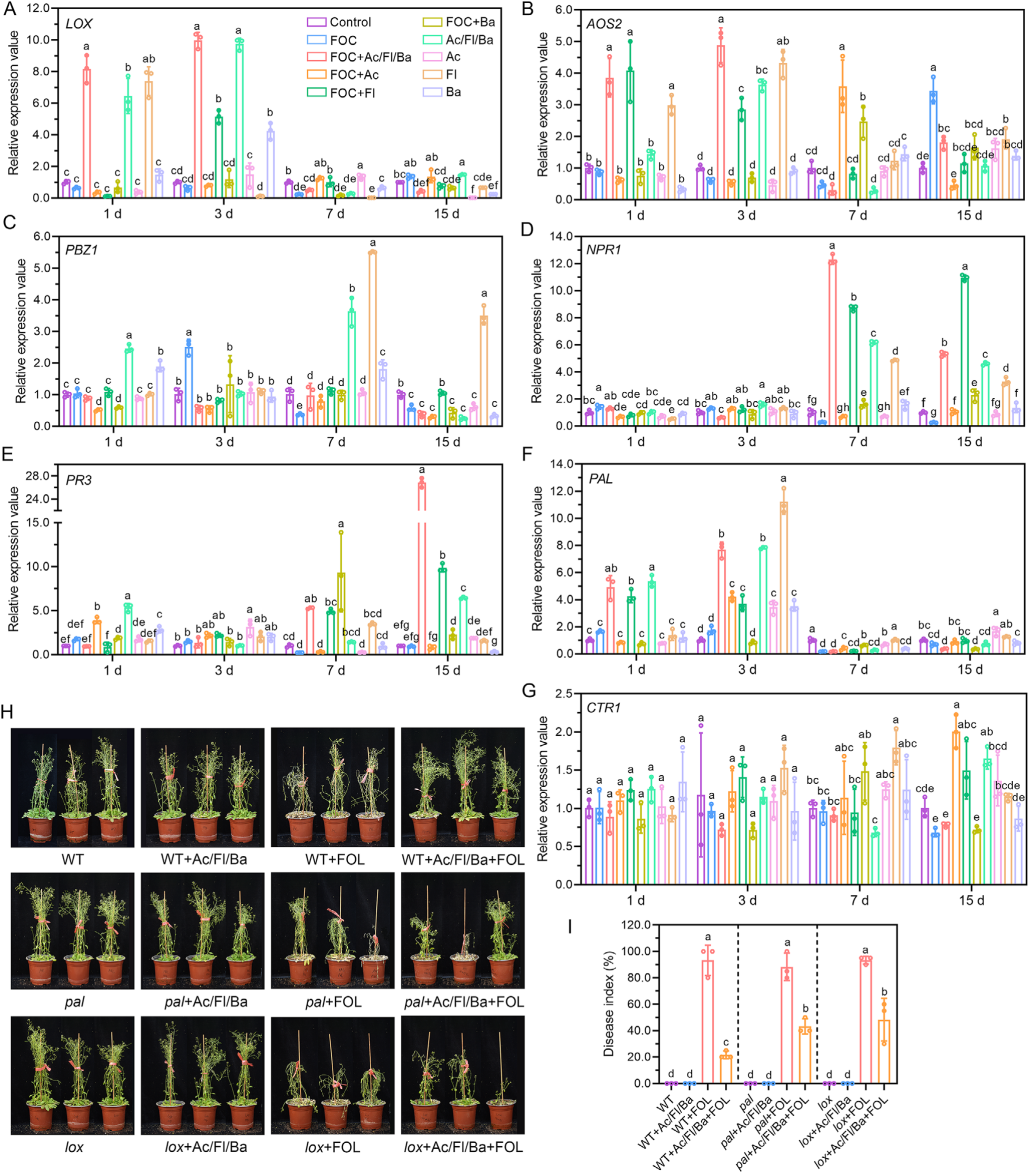
Enhanced immune response in plant against FOC pathogen infection mediated by strain Fl. (A–G) The transcription levels of immune‐related genes in cucumber root tissues at different time after treatment with isolates and consortium, detected using quantitative PCR. Abbreviations: *LOX*, lipoxygenase; *AOS2*, allene oxide synthase; *PBZ1*, probenazole‐inducible protein; *NPR1*, nonexpressor of pathogenesis‐related genes 1; *PR3*, pathogenesis‐related proteins chitinase; *PAL*, phenylalanine ammonia lyase; *CTR1*, constitutive triple response 1. (H and I) Phenotypes and disease index (DI) of wild‐type *Arabidopsis thaliana* plants and mutant lines after treatment with FOL and consortium. Each treatment had three replicates, and values are expressed as mean ± sd. Different letters above the columns indicate significant differences (*p* ≤ 0.05) between treatments according to Tukey's HSD test.

The homozygous *pal* and *lox* mutant lines of *Arabidopsis thaliana* (Figure S25) were used to verify the role of consortium‐induced systemic resistance to overall plant defense. The results showed that FOL‐treated wild‐type Arabidopsis plants had a disease index of 93.3%, whereas those treated with the FOL + Ac/Fl/Ba consortium had a disease index of 21.7%, corresponding to a biocontrol efficacy of 76.7% (Figure 6H,I). This biocontrol efficacy was significantly higher than that in *pal* and *lox* mutant lines (51.0% and 48.2%, respectively) subjected to the same treatment (Figure 6H,I). These results suggests that, in addition to direct antagonism against pathogenic fungi in the soil, the consortium also resists pathogen invasion by inducing systemic immunity in the plant.

### 3.6 Biocontrol of Fusarium Wilt Is *Flavobacterium* Density‐Dependent

To determine the equivalent concentration of *Flavobacterium* in the consortium and elucidate the critical role of the consortium in *Flavobacterium* colonization, pot experiments with different concentrations of Fl treatment were conducted. Compared to the FOC treatment (75.0% disease index), the consortium treatment achieved the lowest Fusarium wilt incidence, with a disease index of only 1.7% and a corresponding biocontrol efficacy of 97.7% (Figure 7A and Figures S26 and S27). In contrast, treatment with Fl at concentrations of 10^8^, 10^7^, and 10^6^ CFU/g soil resulted in significantly higher disease indices of 26.7%, 45.0%, and 65.0%, respectively, with biocontrol efficacies of only 64.4%, 40.0%, and 13.3% (Figure 7A). The cell numbers of Fl in the bulk soil, rhizosphere soil, and root tissues showed a significant positive correlation with its application concentration (Figure 7B–D). Interestingly, although the strain Fl concentration in the consortium was only approximately 10^7^ CFU/g soil, the number of Fl cells detected in this group was significantly higher than that in the 10^8^ CFU/g soil of the Fl treatment (Figure 7B–D). For instance, in the rhizosphere soil, the abundance of Fl in the FOC + Ac/Fl/Ba and Ac/Fl/Ba treatments was 5.4‐ and 3.9‐ fold higher, respectively, than in the FOC + Fl (10^8^ CFU/g soil) and Fl (10^8^ CFU/g soil) treatments (Figure 7C). The results also demonstrated that the microbial consortium significantly enhanced the colonization of Fl in both soil and plant roots. Furthermore, the concentration of Fl in the soil showed a significant negative correlation (*R^2^* = 0.5041, *p* = 7.8e‐14) with the plant disease index, while positive correlating with physiological indicators such as plant height (*R^2^* = 0.4032, *p* = 0.0002234) (Figure 7E,F and Figure S27). The biocontrol effects of different application dosages of Ba against Fusarium wilt were also evaluated. As shown in Figure S28, the AFB consortium also exhibited the best control effect, with a biocontrol efficacy of 91.6% (disease index of 8.3%). The Ba treatment at 10^8^ CFU/g soil showed a control effect comparable to that of the Fl treatment at 10^7^ CFU/g soil (57.1% and 51.2%, respectively), indicating that Fl plays an important role in the suppression of Fusarium wilt by the AFB consortium (Figure S28).

**FIGURE 7:**
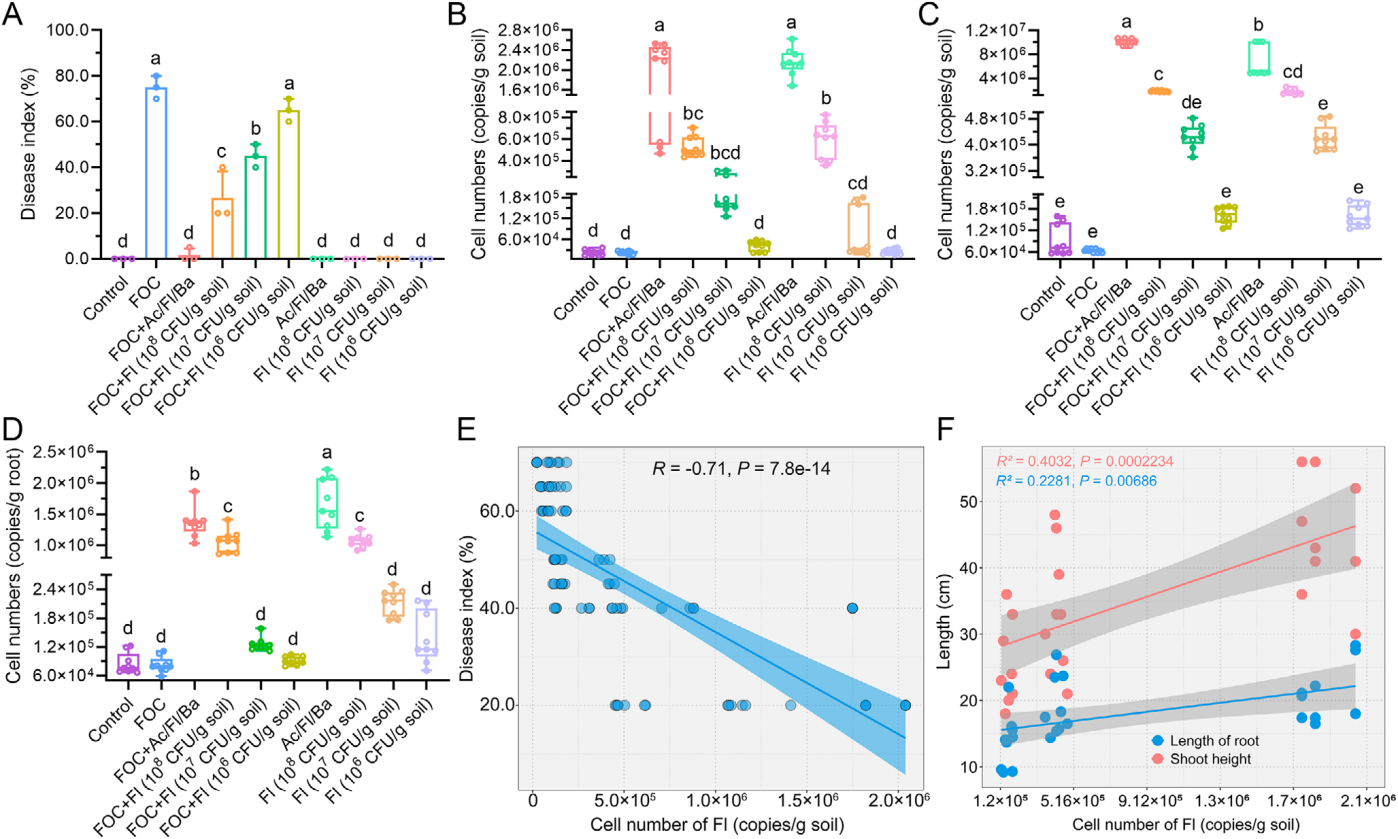
The control efficacy of *Flavobacterium* against Fusarium wilt exhibits a dose‐dependent effect in soil. (A) Statistical analysis of disease indices in cucumber plants treated with different concentrations of strain Fl. Each treatment comprised three biological replicates, with five seedlings per replicate. (B–D) Cell numbers of Fl in bulk soil (B), rhizosphere soil (C), and root tissues (D), determined using qPCR, respectively. (E) Significant negative correlation between *Flavobacterium* abundance and plant disease indices. (F) Significantly positive correlation between *Flavobacterium* abundance and cucumber physiological indices, including root length and shoot height. The qPCR was carried out via QuantStudio 6 Flex system, with three biological replicates per treatment and three technical replicates each. Values are expressed as mean ± sd. Different letters above columns indicate statistically significant differences (*p* ≤ 0.05) based on Tukey's HSD test.

## 4 Discussion

Interactions between plants and microorganisms are complex and tightly coupled. The structure and composition of the soil microbial community determine plant health [35, 36], wherein specific microbial taxa within the microbiome play crucial ecological functions [10]. *Flavobacterium* frequently appears as a keystone species in microbial communities [37]. Moreover, because of their ability to degrade complex polysaccharides and other biomacromolecules, *Flavobacterium* spp. are considered important factors that influence the composition of plant rhizosphere microbial communities [38]. In this study, we report the excellent plant disease control efficacy of a natural microbial consortium collaboratively formed by *Flavobacterium* and other microorganisms isolated from soil treated with predatory myxobacteria, highlighting a novel strategy for endophytic colonization by *Flavobacterium* to enhance plant resistance against pathogen infection.

First, the consortium implementing a “win‐win cooperation” strategy mitigates myxobacterial predation pressure on individual isolates. Predatory microorganisms are also recognized as key soil microbial groups because of their top‐down regulatory effects on microbial communities [18, 39, 40], and their abundance has been used as a key indicator of plant health [41, 42]. In the present study, the *Corallococcus* sp. EGB exhibited excellent predatory activity against the individual isolates (Figure 1A,B). However, compared to single isolates, consortium AFB significantly resisted predation by strain EGB, increasing the number of surviving cells within the consortium (Figure 1). Similar phenomena were observed for the predation activities of other microbial predators (Figure S29). This may be related to the significantly increased biofilm formation observed in the co‐cultured isolates of the consortium (Figure S8). Studies showed that bacterial biofilms could effectively protect against predation by predators [43, 44, 45]. Soil heterogeneity is another important factor that influences the behavior of predatory microorganisms [46]. Nematodes are mainly distributed within macroaggregates and the pores between them, whereas protists, small nematodes, and fungi are located in the pores between microaggregates, and bacteria and archaea are protected within the internal pores of the microaggregates [47]. Therefore, it would be interesting to investigate the spatial distribution of predatory myxobacteria at the soil aggregate scale, and their predatory influence on the succession processes of soil microbial communities.

Second, although community structures dominated by antagonism and competition are more stable than those primarily based on cooperation in large‐scale ecosystems [48], cooperative interactions mediated by the exchange of metabolites may better contribute to the stability of bacterial micro‐consortia [30, 49, 50]. Comparative genomic analyses revealed that over 98% of all sequenced microorganisms lack essential pathways or key genes for amino acid synthesis [50, 51, 52], suggesting the widespread existence of both competitive and cooperative metabolic interactions in nature. In addition to strains NAU1124221 and SQR9, *Achromobacter* and *Flavobacterium* also enhance colony expansion of *Bacillus licheniformis* 14580 and modify the biofilm morphology of *B. megaterium* MS941 (Figure S30), suggesting their potential to cooperate and form microbial consortia within the soil. Trophic interactions are considered the core drivers of microbial community assembly [53]. For example, the prevalence of amino acid auxotrophs not only enhances interdependencies among community members [50], but also increases ecological resilience against environmental stresses [52]. Researchers found that synthetic yeast communities composed of four auxotrophic strains exhibited significantly enhanced resistance to azoles and statin antifungal drugs [52]. A significant reduction in predation by *Corallocuccus* sp. EGB on the three isolates within a minimal consortium also serves as compelling evidence (Figure 1).

Furthermore, metabolic interactions are crucial for shaping microbial community structures; the exchange of metabolites and metabolic byproducts directly influences ecological functional stability within microbial populations [50, 54]. For instance, a microbial consortium formed via cross‐feeding between *B. velezensis* SQR9 and *P. stutzeri* XL272 helped plants alleviate salt stress [30]. Within SQR9 cells, the gene expression of six amino acid biosynthetic pathways is downregulated, reducing the associated metabolic burden [30]. A similar cross‐feeding phenomenon was observed in this study. However, unlike previous findings, except for genes related to the biosynthesis of branched‐chain amino acids, four other amino acid synthesis‐related genes were upregulated in *Bacillus* sp. NAU1124221 (Ba) (Figure 3I,J and Figures S12–S15). Strain NAU112411 (Ac) and Ba are responsible for carbon and nitrogen metabolism, respectively, while the majority of genes in *Flavobacterium* sp. NAU112421 (Fl) were downregulated. Moreover, the differential regulation of *Bacillus* nitrogen metabolism between a previously reported consortium (downregulated) [30] and the current study (upregulated) suggests that microbial metabolic behavior within a consortium undergoes functional adaptations depending on specific cooperative partners. These localized interactions, in turn, may influence the ecological functions of the microbial community [55, 56].

Synergistic biofilm formation represents a common strategy for establishing stable microbial communities [57, 58], facilitating the emergence of community intrinsic properties [59], such as horizontal gene transfer [30], public goods sharing [60], and enhanced resilience to biotic/abiotic stresses [30, 57, 61]. Research indicates that wrinkle‐associated channel networks significantly facilitate fluid transport within biofilms [62]. The formation of wrinkles in the consortium biofilm may facilitate the exchange of nutrients beneath the biofilm and enhance synergistic interactions among the isolates (Figure 3A and Figure S9). Moreover, the biofilm state altered the proportion and distribution of isolates within the microbial consortium. On solid media, biofilm formation significantly promotes colony expansion and acquisition of new nutrients. Metabolic cross‐feeding within the consortium significantly increased the proportions of *Achromobacter* and *Flavobacterium* in the consortium (Figure S4). In the static liquid culture, all three isolates were significantly enriched within the biofilm; however, the number of *Flavobacterium* in the lower liquid medium was significantly decreased (Figure 3D). We speculate that this may be related to a significant decrease in oxygen content within the culture broth.

*Flavobacterium* plays a crucial role in the plant microbiome. Microconsortia formed through cooperation with other microbes provide insights for the development of second‐generation microbial inoculants. For example, *Flavobacterium* not only directly protects plants against infection by various pathogens but also significantly enhances the biocontrol efficacy of other beneficial microbes against phytopathogens [58]. Our study revealed that these three isolates formed stratified biofilms‌ on cucumber root surfaces (Figure 5). Benefiting from this special stratified biofilm structure, strains Fl and Ac were located on the inner side, closely adhering to the plant root surface and facilitating their migration into the root tissues (Figure 4H,I). This behavior, in which endophytic bacteria utilize the layered biofilm structure formed by other rhizosphere microorganisms (e.g., *Bacillus* spp.) to promote colonization of the plant root surface and subsequent migration into the root interior, has never been reported previously and can therefore be regarded as a novel biological strategy for endophytic microbial root entry.

Compared to *Achromobacter* and *Bacillus*, stronger systemically induced resistance was observed in plants treated with *Flavobacterium* (Figure 6A–G), which may play a key role in plant defense against fungal pathogen invasion (Figure 6H,J). Additionally, researches indicate that *Flavobacterium* species can function as biostimulants in various monocot and dicot plants [63, 64, 65]. These effects are likely associated with the production of plant growth‐promoting substances, such as the phytohormone auxin and nitrogen sources [63, 64]. All the three isolates possess the ability to produce IAA and siderophores (Figures S31 and S32), suggesting a potentially promoting effect of the consortium on plant growth. In summary, the stable microbial consortium formed by strains Ac, Fl, and Ba demonstrated excellent biocontrol effects against Fusarium wilt, and exhibited significant application potential as a second‐generation inoculant for the management of soil‐borne fungal diseases.

## Author Contributions

X.F.Y. and Z.L.C. conceptualized the project. X.F.Y., C.S.X., S.Y., J.M.S., and Z.Y.L. performed the experiments. F.W., X.J.L., L.Z. and Y.H. analyzed the data. X.F.Y. wrote the manuscript. Z.K.L., H.Y.Q. and Z.L.C. edited the manuscript.

## Conflicts of Interest

The authors declare no conflicts of interest.

## Supporting information




**Supporting File**: advs77725‐sup‐0001‐SuppMat.docx.

## Data Availability

The raw data files of transcriptomics (GSA: CRA027838) and metabolomics (OMIX: OMIX010985) reported in this paper have been deposited in the Genome Sequence Archive in National Genomics Data Center, China National Center for Bioinformation/Beijing Institute of Genomics, Chinese Academy of Sciences that are publicly accessible at https://ngdc.cncb.ac.cn/gsa.
