# Supplementary material for: Enhanced Endophytic Colonization of Flavobacterium Promotes Plant Resistance to Fusarium Wilt
Source: Adv Sci (Weinh). 2026 Sep 29:e77725. Online ahead of print. doi: 10.1002/advs.77725 (PMC13624321; doi:10.1002/advs.77725)
Supplement: Supplementary file 1 [file ADVS-9999-e77725-s001.docx]

**Enhanced endophytic colonization of *Flavobacterium* promotes plant resistance to Fusarium wilt**

Xianfeng Ye^1^, Changsheng Xu^1^, Shuai Yao^1^, Jiming Shen^1^, Zeyuan Li^1^, Yu Zhang^1^, Fan Wu^1^, Xinjie Li^1^, Yanling Ji^1^, Lei Zhang^1^, Yan Huang^1^, Zhoukun Li^1^, Huayu Qin^2^ and Zhongli Cui^1^*

*^1^Key Laboratory of Agricultural Environmental Microbiology of Ministry of Agriculture and Rural Affairs, Nanjing Agricultural University, Nanjing 210095, China.*

*^2^School of Health Management, Zaozhuang University, Zaozhuang 277160, China.*

***Corresponding authors:** Zhongli Cui

**Corresponding address: ***Key Laboratory of Agricultural Environmental Microbiology of Ministry of Agriculture and Rural Affairs, Nanjing Agricultural University, No.1 Weigang, Nanjing 210095, China.

**Tel.:** +8602584396753, **Fax:** +8602584396753,

**E-mail:** czl@njau.edu.cn

**Materials and methods**

**Identification of amino acid auxotrophy in *Flavobacterium***

*Flavobacterium* sp. NAU112421, along with two other strains NAU1659 and NAU1439, which were also isolated from cucumber rhizosphere soil and belonged to *Flavobacterium*, were cultured in LB medium. Then cells were collected by centrifugation and washed with sterile M9 minimal salts [1] for three times, then resuspended in M9 glucose medium to OD_600_ = 1.0. A 5 μL of cells suspension was added to each well of 48-well plate containing 500 μL of M9 glucose medium supplemented with 10 ppm amino acid. The 48-well plates were incubated at 30 ℃ for 48 h, and the growth of strains was evaluated by measuring the optical density (OD_600_) values using microplate reader SpectraMax i3x. M9 glucose medium supplemented sterile ddH_2_O was used as negative control. Each treatment was repeated three times.

**Predation experiments**

The predatory abilities of strain EGB against each bacterial isolates or consortia were analyzed according to previous reported [2] with a minor modified. The bacterial isolates and strain EGB were collected by centrifugation and resuspended with TPM buffer [2] to OD_600_ of 10 (about 10^9^ CFU/mL) after culturing in LB and LBS medium, respectively. An aliquot of 25 μL of bacterial isolates suspension (bacterial prey) was dropped onto TPM plate, and 5 μL of strain EGB suspension was dropped into the center of the prey cells and incubated at 30 ℃ for 72 h. After that, the bacterial lawn was washed with TPM buffer, and the bacterial suspensions was transferred into 2.0 mL of sterile Eppendorf Safe-Lock Tubes (Eppendorf China, Shanghai, China). This process was repeated three times to ensure that the bacterial isolates from the lawn were completely collected. Then, the number of surviving prey cells from the bacterial lawn was analyzed by serial dilution and spread plating on LB agar plates supplemented with different concentrations of antibiotics. The myxobacterium EGB was unable to grow on LB plates containing 1.0% NaCl. The LB plates spread with the bacterial isolates were incubated at 30 °C for 24 h, after which the colony numbers were counted, and the number of bacterial isolates in the original lawn from the predation experiment was calculated. Each treatment has three replicates and the heat-inactivated strain EGB was used as negative control.

**Antagonistic assay of bacterial isolates against FOC**

The inhibitory effect of the isolates on fungal growth was evaluated using the colony confrontation assay [3] with a minor modification. A 5 mm diameter mycelial plugs were cut from the colonies’ edge and placed in the center of LP medium plates, which was a mixture of equal volumes of LB and PDA medium, after culturing FOC on PDA medium for 5 d. Three bacterial isolates were cultured in LB medium as described above, collected by centrifugation at 8 000×g for 3 min, and then diluted to OD_600_ = 0.2 using LP liquid medium, respectively. Different consortia were obtained by mixing equal volumes of two to three bacterial isolates. Four 5 μL of bacterial isolates or consortia suspensions were spotted at a distance of 1.5 cm from above mycelial plugs. After the suspensions were dried, the plates were inverted and incubated at 25 ℃ for 5 days. Every treatment had three replicates and the sterile ddH_2_O was used as negative control. The diameters of FOC colonies in the medium and on the surface were measured, and the inhibited rates of FOC growth by each bacterial isolates and consortia were calculated. Inhibition rate = [(FOC colony diameter of control group - FOC colony diameter of treatment group)/FOC colony diameter of control group] × 100%.

**Analysis of bacterial isolates co-culture in promoting colony expansion**

To evaluate whether the co-cultivation of bacterial isolates could promote colony expansion, equal volumes of bacterial isolates were mixed at an OD_600_ of 0.2, then 5 μL of cells’ mixtures were spotted on LP agar plates. The plates were incubated at 30 ℃ for 24 h, and the diameters of colonies were measured. Each treatment was replicated three times.

**Quantification of Biofilm formation by the crystal violet method**

Biofilm content of in 96-well plates was quantified using the crystal violet staining method as previous reported [4]. Medium in the sublayer of the biofilm were slowly removed, and the 96-well plates were washed three times with 160 μL sterile ddH_2_O per well to remove residual bacteria. Each well was added with 160 μL of methanol for 15 min of fixation. The methanol was removed, and air dried naturally. Subsequently, 160 μL of 1% crystal violet stain was added to each well, and the plates were incubated at room temperature for 15 minutes. The plates were washed three times with sterile ddH_2_O after stain was removed, and dried at 37 ℃. Finally, 160 μL of ethanol was added into each well, and the plates were placed at 37 ℃ for 30 min. The optical density (OD: 590 nm) of each well was measured using a microplate reader (SpectraMax i3x, Molecular Devices, USA).

**Construction of fluorescence labeling strains**

The plasmid pNW33n-Cm-GFP, stains Bacillus velezensis SQR9 and its mutants, including ΔilvA, ΔilvCH, ΔilvD and ΔleuBCD, were donated by Zhihui Xu, College of resources and environmental sciences, Nanjing Agricultural University, China. The promoter region of the HU family DNA-binding protein gene derived from *Flavobacterium* sp. NAU112421, the replication origin region of the pCP1 plasmid [5], *TaqBFP*, ErmC, and mCherry genes were synthesized by GenScript Biotech Corp. (Nanjing, China) and ligated to the pUC57 plasmid to generate the pUC57-*P_HU_*-*TaqBFP*, pUC57-*ori_pCP1_*, pUC57-ErmC and pUC57-mCherry plasmids, respectively. Chloramphenicol (Cm) and *mCherry* genes were amplified from pNW33n-*Cm-GFP* and pUC57-*mCherry* plasmids, respectively, and then inserted into pBBR1MCS2 plasmid, which was stored at -80 ℃ in our lab, to form pBBR1MCS2-*Cm*-*mCherry* plasmid. ErmC and *ori_pCP1_* genes were cloned and ligated to pUC57-*P_HU_*-*TaqBFP* plasmid to generate *Escherichia coli*–*Flavobacterium* shuttle plasmid pUC57-*ori_pCP1_*-ErmC-*P_HU_*-*TaqBFP*. Finally, the plasmids pNW33n-Cm-GFP, pBBR1MCS2-*Cm*-*mCherry* and pUC57-*ori_pCP1_*-ErmC-*P_HU_*-*TaqBFP* were introduced into cells of *Bacillus*, *Achromobacter* and *Flavobacterium*, respectively, by electroporation as previous described [1, 6]. Transformants were selected by antibiotics and fluorescence of different strains were observed under confocal laser microscopy (CLSM) (Leica TCSSP3, Germany).

**Microscopic observation of biofilms**

Biofilms formed by *Achromobacter* sp. NAU112411, *Flavobacterium* sp. NAU112421 and *Bacillus* sp. NAU1124221 were observed using cryo-scanning electron microscopy (SEM) and laser confocal microscopy to analysis the structural characteristics. Above three fluorescently labeled bacterial isolates or wild strains were inoculated in 6-well plates containing 10 mL LP medium per well at 1% of inoculation, and then the 6-well plates were incubated at 30 ℃ for 48 h. After that, biofilms of each treatment were collected using 1 cm square glass slides, and spread out. Then the area of the biofilm was measured. Mature biofilms were also placed in a freezer at −80 °C for observation of the biofilm structure under SEM (SU8010 SEM, Hitachi, Japan). The protocol used in SEM was following previous reported [7]. Meanwhile, the distribution of three bacterial isolates within biofilms was observed using CLSM.

**DNA and RNA extraction from biofilm formed by coculture of isolates**

The quantities of strains NAU112411, NAU112421 and NAU1121221 were measured in coculture liquid medium and biofilms formed with static coculture were measured, respectively. After 48 h, the cells were collected by centrifugation and stored at -80 ℃ for DNA extraction. For analyzing the quantities of each isolate in the biofilm of coculture, 100 μL of initial inoculum for each strain was transferred into 6 well microtiter plates contained 10 mL LP liquid medium per well. A 100 μm sterile nylon mesh cell strainer (Biologix, Jinan, Shandong, China) and a autoclaved Spectra Mesh Woven Filter with 1.5 cm^2^ (Fisher Scientific, Spectrum 146488) were put inside as previous described [1]. The 6-well plates were incubated at 30 ℃ for 48 h, then inner Filters were taken out from cell strainer and transferred to a 2 mL microcentrifuge tube for DNA or RNA extraction. The cell strainers were removed and the cells in liquid medium were also collected for DNA extraction. Total DNA and RNA of biofilm were extracted using Bacterial DNA Kit and Bacterial RNA Kit (Omega Bio-tek, Inc.), respectively, following the manufacturer’s instructions.

**Microscopic observation of isolates colonization on cucumber roots**

The colonization of cucumber roots by fluorescence-labeled strains were carried out in a modified hydroponic system as previous described [8]. Cucumber (*Cucumis sativus* L.) seeds of “Jingchun 4” were purchased from Jiangsu Academy of Agricultural Sciences (Nanjing, China), then surface-sterilized and germinated following Ye et al. reported [2], then transplanted into 50 mL flasks containing 30 mL of sterile 1/4 sucrose-free Murashige Skoog (MS) medium, and grown in climate chamber at 26 ℃ with a 16/8 h light/dark regimen until the cucumber seedlings have four true leaves. A 100 μL solutions were taken and spread on LB solid medium to check the sterility of hydroponic system before the beginning of colonization assay. The fluorescent strains were cultured and collected as above described, then resuspended with aseptic TPM buffer [2] to OD_600_ = 0.5. The axenically prepared cucumber seedlings were gently washed with sterile ddH_2_O, transferred to a 250 mL flask containing 100 mL of strains’ suspensions, and incubated at same conditions for 48 h. After that, the seedlings were taken out and gently washed with sterile ddH_2_O, cut into 0.3-0.5 cm pieces, and observed under CLSM.

**Chemotactic response of an isolate to cucumber root exudates**

The cucumber root exudates were collected and freeze-dried as previous reported [9], then resuspended in chemotaxis buffer (0.5 g/L KH_2_PO_4_, 1.4 g/L K_2_HPO_4_·3H_2_O, 20 μmol/L EDTA) with final concentrations of 5, 10, 20 and 30 mg/mL. Then root exudates were filtered through a 0.22 μm membrane (Millipore, Merck KGaA, Germany) and stored at -20 ℃ for the chemotaxis assay.

In chemotaxis assay, all isolates were cultured and collected as described above, then resuspended in chemotaxis buffer to OD_600_ = 0.002. A sterile 1.5 mL Eppendorf tube containing 1 mL of bacterial suspension was placed horizontally on a sterile workbench. Then 10 μL pipette tips containing 1 μL of different concentrations of root exudates were inserted horizontally into the bacterial suspensions and left to stand for 45 min. Finally, 1 μL of root exudates were injected into 100 μL chemotaxis buffer and directly coated on R2A medium to measure the number of bacteria.

**Soil sampling and DNA extraction**

Bulk soil more than 3 cm away from the cucumber roots and rhizosphere soil were collected on the 15 and 30 days. Nine samples were randomly selected from fifteen cucumber plants, then three soil samples were randomly chosen and mixed to form one replicate. Sample consisted of 5 g soils were collected at each point at a depth of 5-6 cm. Cucumber roots, stones and visible residues were removed by passing the samples through a 2 mm sieve. Total DNA were extracted from 0.5 g soil samples using the FastDNA® SPIN Kit for soil (MP Biomedicals, Santa Ana, CA) and purified using the DNA Clean-Up^TM^ kit (MOBioLabs, Solana Beach, CA, USA), respectively, following the instruction. The purified DNA was stored at -80 ℃. Meanwhile, Harvested cucumber roots were surface sterilized as previous described [10]. Then, total DNA were extracted from 0.1 g root samples using EZ-10 Spin Column Plant Genomic DNA Purification Kit (Sangon Biotech, Shanghai, China) following the instruction.

**Metabolomic analysis of supernatants from isolates by LC-MS**

Two rounds of extracellular metabolic supernatants were collected and sent to Shanghai Majorbio Bio-pharm Technology Co., Ltd for metabolomics analysis using UHPLC-MS/MS system, which composed of a UHPLC system and an Orbitrap Q Exactive HF-X mass spectrometer (Thermo Fisher, USA). Samples were injected into an Hyperil Gold column (100 × 2.1 mm, 1.9 μm) at a flow rate of 0.2 ml/min with a 16 min linear gradient. The eluents for the positive/negative polarity mode and solvent gradient was set as previous described [1]. The Compound Discoverer 3.0 (CD 3.0, Thermo Fisher) was used to peak alignment, picking, and quantitation of each metabolite after raw data files generated. The molecular formula was predicted by normalized data, which was generated by normalizing all peak intensities with total spectral intensities. Finally, relative quantitative results were obtained by mapping peaks with the mzCloud (https://www.mzcloud.org/) and ChemSpider (http://www.chemspider.com/) database. Each treatment had three replicates.

**Metabolic facilitation was analyzed by growth curve assay**

Strain NAU112411 (Ac), NAU112421 (Fl) and NAU1124221 (Ba) were cultured in LP medium individually for 24 h. Then the cell pellets were removed by centrifugation, and the supernatants were filter sterilized by 0.22 μm sterile filter membrane. 1 mL of freshly cultured bacterial inoculum was inoculated to 100 mL sterile metabolite supernatant. For the coculture system, the metabolite supernatants were obtained by mixing 50 mL of sterile metabolic supernatant from each bacterial isolate, and then 0.5 mL of each freshly cultured bacterial inoculum was inoculated. The flasks were incubated at 30 ℃, and the growth curves were generated by measuring the values of OD_600_ every 1 h using a UV-Visible Spectrophotometer (Evolution 220, Thermo scientific, USA). Each treatment had three replicates.

M9 medium supplemented with sole carbon source was used to search for key metabolites in metabolic syntrophy. The concentration of each carbon source was listed in Table S5. Bacterial isolates were cultured in R2A medium at 30 ℃ for 12 h, respectively. Then the cells were collected and washed with M9 medium three times to remove the residual nutrients. 2 μL of bacterial inoculum (OD_600_ = 1.0) was inoculated to 200 μL M9 medium complemented with sole carbon source in a 10×10 well Honeycomb Microplate. Growth curves of each isolate was plotted by measuring the values of OD_600_ every 30 min at 30 ℃ using Bioscreen C Automated Microbiology Growth Curve Analysis System. Each treatment had three replicates.

**Supplementary Figures and Tables**

**Tables**

**Table S1** Arrangement of pot experiment treatment

| Batch number | Treatment | Objective |
| --- | --- | --- |
| 1 | Control | To compare the biocontrol efficacy of single isolates and microbial consortium against Fusarium wilt |
|  | Ac |  |
|  | Fl |  |
|  | Ba |  |
|  | Ac/Fl/Ba |  |
|  | FOC + Ac |  |
|  | FOC + Fl |  |
|  | FOC + Ba |  |
|  | FOC + Ac/Fl/Ba |  |
|  | FOC |  |
| 2 | Control | To demonstrate the critical role of *Flavobacterium* in the biocontrol of Fusarium wilt |
|  | Ac/Fl/Ba |  |
|  | Fl (10^8^ CFU/g soil) |  |
|  | Fl (10^7^ CFU/g soil) |  |
|  | Fl (10^6^ CFU/g soil) |  |
|  | FOC + Ac/Fl/Ba |  |
|  | FOC + Fl (10^8^ CFU/g soil) |  |
|  | FOC + Fl (10^7^ CFU/g soil) |  |
|  | FOC + Fl (10^6^ CFU/g soil) |  |
|  | FOC |  |

Note: Control indicates no treatment (no FOC pathogen and other bacterial isolates treatment); “Ac”, “Fl” and “Ba” represent *Achromobacter* sp. NAU112411, *Flavobacterium* sp. NAU112421 and *Bacillus* sp. NAU1124221, respectively; FOC represents FOC pathogen treatment only; Ac/Fl/Ba indicates the group treated with strains NAU112411, NAU112421 and NAU1124221; FOC + Ac/Fl/Ba represents the group treated with FOC pathogen, NAU112411, NAU112421 and NAU1124221.

**Table S2** Primers for defense response genes of cucumber used in qRT-PCR

| Gene names | Function | Primers’ name | Sequence 5’-3’ |
| --- | --- | --- | --- |
| *Actin* | Actin | Actin-F | TCCACGAGACTACCTACAACTC  GCTCATACGGTCAGCGAT |
|  |  | Actin-R |  |
| *NPR1* | Nonexpresser of Pathogenesis-Related genes 1 | Npr-F | TTACTGATAAGGGCAAGAAGGCC  AAAGTTCACAAAGAGCAGGATGG |
|  |  | Npr-R |  |
| *PR3* | Chitinase | Pr-F | TGGTCACTGCAACCCTGACA  AGTGGCCTGGAATCCGACT |
|  |  | Pr-R |  |
| *LOX1* | Lipoxygenase | Lox-F | AAGGTTTGCCTGTCCCAAGA  TGAGTACTGGATTAACTCCAGCCAA |
|  |  | Lox-R |  |
| *CTR1* | Threonine-protein Kinase | Ctr-F | AAACACGTCGGATAAATATGGCTT  CATCCATTCAGGCGTTCCAG |
|  |  | Ctr-R |  |
| *PAL1* | Phenylalanine Ammonia-lyase | Pal-F | ATGGAGGCAACTTCCAAGGA  CCATGGCAATCTCAGCACCT |
|  |  | Pal-R |  |
| *PBZ1* | major allergen Pru ar 1-like | Pbz-F | GGTTCCTCCAGCTAAATT  ATGATGCTTCGTCCACTA |
|  |  | Pbz-R |  |
| *AOS2* | Allene Oxide Synthase | Aos-F | CCGAATTACCAAATACAACT  ATGGGTCCAAATAAGCAC |
|  |  | Aos-R |  |

**Table S3** Oligonucleotide primers for quantification of bacterial isolates and FOC pathogen.

| Strain | Primers’ name | Sequence (5’-3’) |
| --- | --- | --- |
| *Achromobacter* sp. NAU112411 | Ac-F | TTGCCTGTGCGCTTTCGG |
|  | Ac-R | CCTGGCACTCGTGGATCTGG |
| *Flavobacterium* sp. NAU112421 | Fl-F | GCGACAATAACTTCAGG |
|  | Fl-R | GTCATCCAAGCACCAA |
| *Bacillus* sp. NAU1124221 | Ba-F | GCGTGCCTGGACTCAT |
|  | Ba-R | GCCGCTTACTCCTCTAT |
| FOC | FOC-F | AAACGAGCCCGCTATTTGAG |
|  | FOC-R | TATTTCCTCCACATTGCCATG |

**Table S4** Primers were used to validate the accuracy of transcriptome sequencing for isolates in coculture.

| Gene names | Primers’ name | Sequence 5’-3’ |
| --- | --- | --- |
| *gapdh1* | Gapdh-F | AGAGGTTGTGGCTGGTG |
|  | Gapdh-R | TTGAAATAATGACGTGGC |
| *ilvE* | IlvE-F | CAGGGTGTTGCTGTTCC |
|  | IlvE-R | TCGTCGCCATAATAGGAG |
| *trpB* | TrpB-F | TTATCCGCAAGTGGTCC |
|  | TrpB-R | GCCCGAACTCATCCTG |
| *trpD* | TrpD-F | ATGGCAATCGCTCTGTC |
|  | TrpD-R | TGACGCTTCGCCTGTA |

**Table S5** Information of the compounds and concentration provided as sole carbon source in M9 medium

| Compounds | CAS no. | Molecular formula | Source | Purity | Concentration | Microbial growth detection (OD_600_) | | |
| --- | --- | --- | --- | --- | --- | --- | --- | --- |
|  |  |  |  |  |  | Ac | Fl | Ba |
| Glucose | 50-99-7 | C_6_H_12_O_6_ | Solarbio | ≥99.8% | 10.0 g/L | + | + | + |
| Deoxyadenosine | 958-09-8 | C_10_H_13_N_5_O_3_ | Solarbio | ≥98.0% | 3.0 g/L | / | / | - |
| Adenosine monophosphate | 130-49-4 | C_10_H_14_N_5_O_7_P | Aladdin | ≥98.0% | 10.0 g/L | / | / | - |
| Thymidine | 50-89-5 | C_10_H_14_N_2_O_5_ | Aladdin | ≥99.0% | 10.0 g/L | / | / | 0.09±0.01 |
| Acetyl-L-tyrosine | 537-55-3 | C_11_H_13_NO_4_ | Merck | ≥98.0% | 10.0 g/L | 1.17±0.05 | / | / |
| N-Acetyl-L-phenylalanine | 2018-61-3 | C_11_H_13_NO_3_ | Aladdin | ≥99.0% | 10.0 g/L | 1.27±0.02 | / | / |
| 6-Hydroxynicotinic acid | 5006-66-6 | C_6_H_5_NO_3_ | Aladdin | ≥99.0% | 2.0 g/L | 0.52±0.02 | / | / |
| Dihydrocoumarin | 119-84-6 | C_9_H_8_O_2_ | Merck | ≥99.0% | < 0.1 mg/L | - | / | / |
| 4-Hydroxybenzaldehyde | 123-08-0 | C_7_H_6_O_2_ | Merck | ≥98.0% | 10.0 g/L | - | / | / |
| 3-Hydroxy-2-methylglutarate | 1189155-79-0 | C_7_H_12_O_5_ | Merck | ≥95.0% | 10.0 g/L | - | / | / |
| N-Acetyl-L-glutamic acid | 1188-37-0 | C_7_H_11_NO_5_ | Merck | ≥99.0% | 10.0 g/L | 1.30±0.06 | / | / |
| Hydroxyprolyl-Isoleucine | 51926-51-3 | C_11_H_20_N_2_O_3_ | Aladdin | ≥98.0% | 10.0 g/L | - | / | / |
| Cinnamic acid | 140-10-3 | C_9_H_8_O_2_ | Aladdin | ≥99.0% | < 0.1 mg/L | - | / | / |
| Juglone | 481-39-0 | C_10_H_6_O_3_ | Merck | ≥97.0% | Dissolve to saturation | / | / | 0.09±0.00 |
| Inosine | 58-63-9 | C_10_H_12_N_4_O_5_ | Merck | ≥99.0% | 10.0 g/L | / | / | - |
| N2-Acetyl-L-ornithine | 6205-8-9 | C_7_H_14_N_2_O_3_ | MedBio | ≥98.0% | 10.0 g/L | / | / | - |
| Glycylproline | 704-15-4 | C_7_H_12_N_2_O_3_ | TargetMol | ≥95.0% | 10.0 g/L | / | / | 0.04±0.00 |
| 5-Hydroxylysine | 1190-94-9 | C_6_H_14_N_2_O_3_ | InnoChem | ≥95.0% | 10.0 g/L | / | / | - |
| N-Acetyl-7-O-acetylneuraminic acid | 18529-63-0 | C_13_H_21_NO_10_ | Selleck | ≥99.0% | 10.0 g/L | / | / | - |
| 1-(2,4-Dichlorophenyl)-2-(1-imidazolyl) ethanol | 24155-42-8 | C_11_H_10_Cl_2_N_2_O | Aldrich | ≥98.0% | Dissolve to saturation | / | / | - |
| Deoxycytidine | 951-77-9 | C_9_H_13_N_3_O_4_ | Merck | ≥99.0% | 10.0 g/L | / | - | / |
| pterine | 2236-60-4 | C_6_H_5_N_5_O | Merck | ≥95.0% | 10.0 g/L | - | - | / |
| Dihydro-4,4-Dimethyl-2,3-furandione | 13031-04-4 | C_6_H_8_O_3_ | Merck | ≥97.0% | 10.0 g/L | / | - | / |
| N-alpha-Acetyl-L-arginine | 155-84-0 | C_8_H_16_N_4_O_3_ | Merck | ≥98.0% | 10.0 g/L | / | - | / |
| Embelin | 550-24-3 | C_17_H_26_O_4_ | Merck | ≥98.0% | Dissolve to saturation | / | - | / |
| Docosadienoic acid | 17735-98-7 | C_22_H_40_O_2_ | Aladdin | ≥98.0% | Dissolve to saturation | / | - | / |
| 2-(Octadecyloxy)ethanol | 2136-72-3 | C_20_H_42_O_2_ | Aladdin | ≥98.0% | Dissolve to saturation | / | - | / |
| lactic acid | 10326-41-7 | C_3_H_6_O_3_ | MCE | ≥95.0% | 10.0 g/L | 1.23±0.01 | / | / |
| Adipic acid | 124-04-9 | C_6_H_10_O_4_ | Merck | ≥99.0% | 10.0 g/L | 0.97±0.03 | / | / |
| Citramalic acid | 2306-22-1 | C_5_H_8_O_5_ | OwowLife | ≥95.0% | 10.0 g/L | 1.37±0.05 | / | / |
| Dihydroxyacetone | 96-26-4 | C_3_H_6_O_3_ | Acmec | ≥98.0% | 10.0 g/L | - | / | / |
| 3-Oxohexanoic acid | 4380-91-0 | C_6_H_10_O_3_ | Macklin | ≥95.0% | 10.0 g/L | - | / | / |
| 3-Methyl-2-oxovaleric acid | 1460-34-0 | C_6_H_10_O_3_ | Macklin | ≥98.0% | 10.0 g/L | - | / | / |
| 5'-Deoxy-5'-(methylthio)adenosine | 2457-80-9 | C_11_H_15_N_5_O_3_S | Merck | ≥98.0% | Dissolve to saturation | / | / | - |
| 2-Deoxy-2,3-dehydro-N-acetylneuraminic acid | 24967-27-9 | C_11_H_17_NO_8_ | Merck | ≥98.0% | 2.5 g/L | / | / | - |
| N-Acetyl-L-methionine | 65-82-7 | C_7_H_13_NO_3_S | Merck | ≥98.5% | 10.0 g/L | / | - | / |
| N-Methyl-a-aminoisobutyric acid | 2566-34-9 | C_5_H_11_NO_2_ | Merck | ≥97.0% | 10.0 g/L | / | - | / |
| Berberine | 2086-83-1 | C_20_H_17_NO_4_ | Aladdin | ≥99.0% | 1.0 g/L | / | - | / |
| 1-heptadecanoyl-glycero-3-phosphate | 50930-23-9 | C_20_H_41_O_7_P | Aladdin | ≥99.0% | 10.0 g/L | / | - | / |
| Glycyl-Phenylalanine | 721-66-4 | C_11_H_14_N_2_O_3_ | Aladdin | ≥98.0% | 10.0 g/L | / | - | / |
| Alanyl-Proline | 13485-59-1 | C_8_H_14_N_2_O_3_ | Aladdin | ≥96.0% | 10.0 g/L | / | 0.34±0.03 | / |
| Pro Pro Ile Leu | / | / | GenScript | ≥98.0% | 5.0 g/L | / | / | 0.06±0.00 |
| Ile Pro Val | / | / | GenScript | ≥98.0% | 5.0 g/L | / | / | 0.04±0.00 |
| Gly Asp Leu | / | / | GenScript | ≥98.0% | 5.0 g/L | / | 0.08±0.00 | / |
| Thr Leu Pro Pro Gly | / | / | GenScript | ≥98.0% | 5.0 g/L | / | - | / |
| Asn Glu Thr Leu |  |  | GenScript | ≥98.0% | 5.0 g/L | / | - | / |

Note: 10 g/L glucose was added to M9 medium as a positive control, and M9 medium was used as negative control. "+" indicates that the strain can grow normally when the substance serves as the sole carbon source; "-" indicates that the strain cannot grow normally when the substance serves as the sole carbon source; "/" indicates that the corresponding compound was not tested as the sole carbon source for strain growth.

**Table S6** Oligonucleotide primers for mutants’ analysis

| Mutants | Gene ID | SALK number | Primers’ name | Sequence (5’-3’) |
| --- | --- | --- | --- | --- |
| *pal* | AT2G37040 | SALK_000357C | 37040-LP | GTGTCGCACTTCAGAAGGAAC |
|  |  |  | 37040-RP | TAACGGATCCATCTCGTGAAG |
| *lox* | AT1G17420 | SALK_147830C | 17420-LP | TAGCGTTCAACATAGGTTCGG |
|  |  |  | 17420-RP | ACGCATGGTTAAGAGATGACG |
|  |  |  | BP | ATTTTGCCGATTTCGGAAC |

**Figures**

**Figure S1** Phylogenetic tree analysis based on 16S rRNA gene sequences was performed for the three bacterial isolates obtained from the microbial consortium. The selection of strains used in phylogenetic tree was determined based on the blast results of 16S rRNA gene sequences of the three bacterial isolates in the EzBioCloud database (<https://eztaxon-e.ezbiocloud.net/>). And MEGA 11 (version 11.0.13) software was employed to construct the phylogenetic tree.

**Figure S2** Analysis of colony expansion capability on LP medium by three isolates and microbial consortium formed through co-cultivation of different bacterial strains. Colony diameters are shown as mean ± sd of six replicates (three biological replicates, each measured in duplicate from different angles). Different letters above columns indicate significant differences among treatments at *P* ≤ 0.05 based on Tukey’s Honestly Significant Difference (HSD) test. “Ac”, “Fl” and “Ba” represent *Achromobacter* sp. NAU112411, *Flavobacterium* sp. NAU112421, and *Bacillus* sp. NAU1124221, respectively.

**Figure S3** Analysis of the growth of *Flavobacterium* in M9 glucose minimal medium supplemented with 10 ppm of various amino acids. Strains NAU1439 and NAU1659 are both *Flavobacterium* isolates originating from cucumber rhizosphere soil. **A**, **B** and **C** show the OD_600_ values of strains NAU112421 (Fl), NAU1439 and NAU1659, respectively, after 48 h of cultivation in M9 glucose minimal medium containing 20 amino acids or their mixture (Mix). **D** shows the growth status of the three strains in 96-well microplates containing above medium. The data were presented as the mean ± standard deviation from the three biological replicates. Different letters above columns indicate significant differences among treatments at *P* ≤ 0.05 based on Tukey’s HSD test.

**Figure S4** The cell numbers of specific bacterial isolates after monoculture and coculture. **A, B** and **C** represent the cell numbers of Ac, Fl and Ba, respectively. The bacterial isolates and different consortiums were cultured on M9 solid medium containing 1.0% glucose for 5 days. Then, based on the differences in antibiotic susceptibility and colony morphology among the bacterial isolates, each specific bacterial isolate was enumerated by the dilution spread plate method. Ac/Ba, Fl/Ba, and Ac/Fl/Ba indicate the cocultures of the corresponding bacterial isolates. The data were presented as the mean ± standard deviation from the three biological replicates. Different letters above columns indicate significant differences among treatments at *P* ≤ 0.05 based on Tukey’s HSD test. “Ac, Fl and Ba” represent *Achromobacter* sp. NAU112411, *Flavobacterium* sp. NAU112421 and *Bacillus* sp. NAU1124221, respectively.

**Figure S5** The isolates within microbial consortium collaborate via metabolic cross-feeding. **A**, Schematic of the experiment showing growth stimulation of other isolates by metabolites from a donor isolate. Strain NAU112411 (Ac) was cultured in M9 glucose medium for 3 d till the glucose could not be detected. The spend supernatant was collected and filtered, then, used as the growth medium for other isolates. **B** and **C** represent OD_600_ absorbance of cell cultures and cell numbers of isolates in cultures at day 4 in panel (**A**), respectively. The cell numbers of isolates after 4 d of cultivation in the metabolite were determined by the serial dilution spread plate method. **D** and **F** represent volcano plots showing differentially abundant metabolites in strain NAU1124221 (Ba) and NAU112421 (Fl), respectively, after 4 d of culture in spent supernatant from strain NAU112411 (Ac). **E** and **G**, Metabolic profiles of the spent medium experiment. “Ac” indicated the spent supernatant of strain NAU112411 (Ac) grown on M9 glucose, “Ac_Ba” indicated the spent medium of strain NAU1124221 (Ba) grown on cell-free filtrate of strain NAU112411 (Ac). “Ac_Fl” indicated the spent medium of strain NAU112421 (Fl) grown on cell-free filtrate of strain NAU112411 (Ac). Red arrows indicate substrates utilized by strains NAU1124221 in the spent M9 glucose supernatant from strain NAU112411. **H** represent growth curves of strain NAU1124221 in the M9 minimal medium supplemented with the tested compounds as the sole carbon source. All treatments were performed with three biological replicates. Values are expressed as mean ± standard deviation. For panels B and C, absorbance and qPCR quantification of bacterial isolates are expressed as mean ± sd from three biological replicates, each with three technical replicates (total n = 9). Different letters above columns indicate significant differences among treatments according to Tukey’s HSD test at *P* ≤ 0.05.

**Figure S6** The isolates within microbial consortium collaborate via metabolic cross-feeding. **A**, Schematic of the experiment showing growth stimulation of other isolates by metabolites from a donor isolate. Strain NAU112421 (Fl) was cultured in M9 glucose medium supplemented with 15 ppm methionine for 13 d till the glucose could not be detected. The spend supernatant was collected and filtered, then, used as the growth medium for other isolates. **B** and **C** represent OD_600_ absorbance of cell cultures and cell numbers of isolates in cultures at day 4 in panel (**A**), respectively. The cell numbers of isolates after 4 d of cultivation in the metabolite were determined by the serial dilution spread plate method. **D** and **F** represent volcano plots showing differentially abundant metabolites in strain NAU112411 (Ac) and NAU1124221 (Ba), respectively, after 4 d of culture in spent supernatant from strain NAU112421 (Fl). **E** and **G**, Metabolic profiles of the spent medium experiment. “Fl” indicated the spent supernatant of strain NAU112421 (Fl) grown on M9 glucose, “Fl_Ac” indicated the spent medium of strain NAU112411 (Ac) grown on cell-free filtrate of strain NAU112421 (Fl). “Fl_Ba” indicated the spent medium of strain NAU1124221 (Ba) grown on cell-free filtrate of strain NAU112421 (Fl). Red arrows indicate substrates utilized by strains NAU112411 and NAU1124221 in the spent M9 glucose supernatant from strain NAU112421. **H** and **J** represent growth curves of strain NAU112411 and NAU1124221, respectively, in the M9 minimal medium supplemented with the tested compounds as the sole carbon source. All treatments were performed with three replicates. Values are expressed as mean ± standard deviation. For panels B and C, absorbance and qPCR quantification of bacterial isolates are expressed as mean ± sd from three biological replicates, each with three technical replicates (total n = 9). Different letters above columns indicate significant differences among treatments according to Tukey’s HSD test at *P* ≤ 0.05.

**Figure S7** Determination of amino acid concentrations in the supernatant of isolates monocultured and cocultured in M9 glucose medium using UPLC-MS. The data were presented as the mean ± standard deviation from three replicates. Different letters above columns indicate significant differences among treatments at *P* ≤ 0.05 based on Tukey’s HSD test. “Ac, Fl and Ba” represent *Achromobacter* sp. NAU112411, *Flavobacterium* sp. NAU112421 and *Bacillus* sp. NAU1124221, respectively.

**Figure S8** The biofilm formation of single isolates and consortium cultured in LP liquid medium was assessed. The morphology of biofilms formed by bacterial isolates and consortia cultured in 96-well plates containing 200 μL/well of LP medium was observed. And the biofilms formed by single isolates or consortia were quantified using crystal violet staining. Each treatment was performed in triplicate, and values are expressed as mean ± standard deviation (SD). Different letters above columns indicate significant differences among treatments according to Tukey’s HSD test at *P* ≤ 0.05. “Ac, Fl and Ba” represent *Achromobacter* sp. NAU112411, *Flavobacterium* sp. NAU112421, and *Bacillus* sp. NAU1124221, respectively.

**Figure S9** Scanning electron microscopy (SEM) image of the microstructure of the biofilm formed by Ba or microbial consortium. “Ac, Fl and Ba” represent *Achromobacter* sp. NAU112411, *Flavobacterium* sp. NAU112421, and *Bacillus* sp. NAU1124221, respectively.

**Figure S10** Analysis of cell numbers of strains Ac and Fl in monoculture and coculture in LP liquid medium. **A**, Growth status of isolates Ac and Fl in a 6-well plate containing LP liquid medium. **B**, Cell numbers of strains Ac and Fl in monoculture and coculture were determined by the serial dilution spread plate method. All treatments were performed with three replicates. Values are expressed as mean ± sd. Different letters above columns indicate significant differences among treatments according to Tukey’s HSD test at *P* ≤ 0.05.

**Figure S11** The proportion and numbers of significantly regulated genes in KEGG pathways within the cells of *Achromobacter* sp. NAU112411 (Ac), *Flavobacterium* sp. NAU112421 (Fl), and *Bacillus* sp. NAU1124221 (Ba) after 24 h of coculture in LP liquid medium.

**Figure** **S12** Relative transcriptional levels of genes involved in tryptophan biosynthesis. Color indicates the mean relative fold change of mRNA in each strain during coculture versus monoculture.

**Figure S13** Relative transcriptional levels of genes involved in arginine biosynthesis. Color indicates the mean relative fold change of mRNA in each strain during coculture versus monoculture.

**Figure S14** Relative transcriptional levels of genes involved in histidine biosynthesis. Color indicates the mean relative fold change of mRNA in each strain during coculture versus monoculture.

**Figure S15** Relative transcriptional levels of genes involved in methionine biosynthesis. Color indicates the mean relative fold change of mRNA in each strain during coculture versus monoculture.

**Figure S16** The transcriptional levels of selected genes significantly regulated during coculture were detected using quantitative real-time PCR (qRT-PCR) based on 2^-ΔΔCT^ method, to validate the accuracy of the RNA-seq results. An *ilvE* gene (significantly downregulated in branched-chain amino acid synthesis) and *trpB*/*trpD* genes (significantly upregulated in tryptophan synthesis) from Ba were selected for validation. The results demonstrated that, compared to Ba monoculture, the relative expression levels of *ilvE*, *trpB* and *trpD* in coculture were 0.72-, 2.59-, and 3.14-fold of the control group, respectively. These changes demonstrated similar trends to the RNA-seq data, confirming the accuracy of the transcriptome sequencing results. Each treatment has three replicates, and the data are represented as the mean ± standard deviation. Different letters above columns indicate significant differences among treatments according to Tukey’s HSD test at *P* ≤ 0.05. “Ac, Fl and Ba” represent *Achromobacter* sp. NAU112411, *Flavobacterium* sp. NAU112421 and *Bacillus* sp. NAU1124221, respectively.

**Figure S17** Biofilms’ formation of strain SQR9 and its mutants in monoculture or coculture with Ac and Fl in Msgg medium after 24 h. **A**, Quantification of biofilm biomass via crystal violet staining for strain SQR9 and mutants in monoculture or coculture. **B**, The cell numbers in the liquid medium beneath were determined by the serial dilution spread plate method for strain SQR9 and its mutants, either in monoculture or in coculture with Ac and Fl. All treatments were performed with three replicates. Values are expressed as mean ± standard deviation. Different letters above columns indicate significant differences among treatments according to Tukey’s HSD test at *P* ≤ 0.05.

**Figure S18** Biofilm formation and cell numbers of strain SQR9 and its mutants in LP medium supplemented with 20 ppm branched chain amino acids (BCAAs) after 24 h. **A**, Quantification of biofilm biomass via crystal violet staining for strain SQR9 and its mutants after 24 h of incubation. **B**, The cell numbers beneath the biofilm formed by strain SQR9 and its mutants were determined by dilution spread plate method in LP medium with and without the supplementation of 20ppm BCAAs. All treatments were performed with three replicates. Values are expressed as mean ± standard deviation. Different letters above columns indicate significant differences among treatments according to Tukey’s HSD test at *P* ≤ 0.05.

**Figure S19** Growth status of cucumber plants treated with single isolates and Ac/Fl/Ba microbial consortium. “Ac, Fl and Ba” represent *Achromobacter* sp. NAU112411, *Flavobacterium* sp. NAU112421, and *Bacillus* sp. NAU1124221, respectively.

**Figure S20** Physiological parameters, including shoot height (**A**), length of root (**B**), average stem diameter (**C**) and the biggest diameter of leaf (**D**), were evaluated in plants treated with single isolates and Ac/Fl/Ba microbial consortium. Each treatment consisted of five pots, with three cucumbers per pot. The values were expressed as mean ± standard deviation. Different letters above columns indicate significant differences among treatments at *P* ≤ 0.05 based on Tukey’s HSD test. “Ac, Fl and Ba” represent *Achromobacter* sp. NAU112411, *Flavobacterium* sp. NAU112421 and *Bacillus* sp. NAU1124221, respectively.

**Figure S21** Strains NAU112411 (Ac), NAU112421 (Fl) and NAU1124221 (Ba) were fluorescently labeled with mCherry, BFP and GFP proteins, respectively. The images show the cellular morphology of the fluorescently labeled strains observed under a Leica DMi8 inverted fluorescence microscope (Leica Microsystems, Wetzlar, Germany). “BFI” represents bright-field illumination, and "FFI" indicates fluorescence-field illumination. Bar = 10 μm.

**Figure S22** Colonization observation of strains NAU112411 (Ac), NAU112421 (Fl) and NAU1124221 (Ba) in cucumber roots. *Achromobacter* sp. NAU112411 (mCherry-labeled) and *Flavobacterium* sp. NAU112421 (BFP-labeled) migrated into roots from lateral root emergence sites and primarily colonized the intercellular spaces of root cortex cells. GFP-labeled *Bacillus* sp. NAU1124221 could not be detected within the plant roots, indicating that strains NAU112111 and NAU112421 from the micro-community function as cucumber endophytes, while NAU1121221 cannot colonize cucumber roots. Bar = 75 μm.

**Figure S23** Chemotaxis analysis of bacterial isolates from microbial consortium toward different concentrations of cucumber root exudates. The numbers of bacterial isolates were determined by the dilution spread plate method. All treatments were performed with three replicates. Values are expressed as mean ± standard deviation. Different letters above bars indicate significant differences among treatments according to Tukey’s HSD test at *P* ≤ 0.05.

**Figure S24** Analysis of the utilization ability of strains NAU112411 (Ac), NAU112421 (Fl) and NAU1124221 (Ba) for the compounds identified from cucumbers’ exudates. Three isolates were cultured overnight in R2A medium. The bacterial cells were then collected by centrifugation at 12000 rpm for 1 min, and washed three times with M9 medium to remove residual R2A medium from the cell surfaces. The cell pellets were resuspended in M9 medium to an OD_600_ of 1.0. Subsequently, a 1% inoculum of isolates was transferred into M9 medium containing the test compound as the sole carbon source. After 48 h of incubation, the OD_600_ value of cell cultures was measured. Each treatment has three replicates, and the colors in the heatmap represent the average of three measurements. The composition of root exudates is based on previously reported reference [11].

**Figure S25** Genotypic verification of T-DNA insertion Arabidopsis mutants purchased from AraShare Company ([www.arashare.cn](http://www.arashare.cn)). Primers LP and RP flank the genomic sequence of the insertion site, while LB represents left border primer of the T-DNA insertion. Using wild-type (WT) Arabidopsis genomic DNA as template, the LP/RP primer pair theoretically amplifies a product of approximately 1000-11000 bp, whereas no amplification occurs in mutants. With mutant Arabidopsis genomic DNA as template, the LB/RP primer pair theoretically yields a ~500 bp product, with no amplification in wild-type plants. The results in the figure demonstrate that both *pal* and *lox* mutants are homozygous Arabidopsis mutants, and suitable for subsequent experiments.

**Figure S26** Evaluation of the efficacy of *Flavobacterium* treatments at different concentrations on the suppression of cucumber Fusarium wilt. “Ac, Fl and Ba” represent *Achromobacter* sp. NAU112411, *Flavobacterium* sp. NAU112421 and *Bacillus* sp. NAU1124221, respectively.

**Figure S27** Physiological parameters, including shoot height (**A**), root length (**B**), average stem diameter (**C**), and the biggest leaf diameter (**D**), were measured in cucumber plants treated with *Flavobacterium* at different concentrations. Each treatment has five pots, with three cucumbers every pot. The data were expressed by mean ± standard deviation. Different letters above columns indicate significant differences among treatments at *P* ≤ 0.05 based on Tukey’s HSD test. “Ac, Fl and Ba” represent *Achromobacter* sp. NAU112411, *Flavobacterium* sp. NAU112421 and *Bacillus* sp. NAU1124221, respectively.

**Figure S28** Evaluation of the effect of *Bacillus* sp. NAU1124221 (Ba) application dosage on the control of cucumber Fusarium wilt. **A**, Growth situation and disease symptoms of cucumber plants in different treatment groups. Each treatment comprised three biological replicates, with five seedlings per replicate. **B**, Disease index of cucumber in different treatment groups. **C-F**, Determination of physiological parameters of cucumber, including plant height (**C**), root length (**D**), average stem diameter (**E**), and diameter of the largest leaf (**F**). **G**, Quantification of *Bacillus* abundance in soil from the Ba-treated groups by quantitative qPCR. **H**, Quantification of *Fusarium oxysporum* f. sp. *cucumerinum* (FOC) cell numbers in soil from the FOC spore-treated groups by quantitative qPCR. The qPCR was carried out via QuantStudio™ 6 Flex system, with three biological replicates per treatment and three technical replicates each. Values are expressed as mean ± sd. Different letters above columns indicate statistically significant differences (*P* ≤ 0.05) based on Tukey’s HSD test.

**Figure S29** The predation efficacy of other predatory microorganisms, including *Microvirga* sp. MC18, *Cystobacter* sp. CC21 and *Archangium* sp. AC19, on single isolates and microbial consortium. “-’’ indicates untreated colonies, while “+” denotes colonies treated with predatory microorganisms (**A**). The red dashed line marks the diffusion boundary of strain EGB from the colony center to the edge due to predation. **B**, **C** and **D** represent the residual quantities of *Achromobacter* (Ac), *Flavobacterium* (Fl) and *Bacillus* (Ba) in colonies after predation, respectively. The surviving isolates on the bacterial lawn after predation were counted by the dilution spread plate method. Each treatment was performed in triplicates, and data expressed as mean ± standard deviation. Different lowercase letters above columns indicate significant differences among treatments at *P* ≤ 0.05 based on Tukey’s HSD test.

**Figure S30** Analysis of colony expansion of strain NAU112411 (Ac), NAU112421 (Fl) and species from other *Bacillus* genera co-cultured on LP medium. Values are expressed as mean ± standard deviation from six replicates. Different letters above bars indicate significant differences among treatments according to Tukey’s HSD test at *P* ≤ 0.05.

**Figure S31** The IAA production of isolates from the original microbial consortium in R2A medium without (**A**) and with (**B**) tryptophan. **C**, IAA content in the culture supernatant of each isolate were measured using the method as described[12]. Each treatment was performed in triplicates, and data expressed as mean ± standard deviation. Different lowercase letters above columns indicate significant differences among treatments at *P* ≤ 0.05 based on Tukey’s HSD test. “Ac, Fl and Ba” represent *Achromobacter* sp. NAU112411, *Flavobacterium* sp. NAU112421, and *Bacillus* sp. NAU1124221, respectively.

**Figure S32** Siderophore production by isolates derived from the original microbial consortium in Fe iron-free R2A medium (**A**). Siderophore content in the culture supernatant of each isolate was quantified using the chrome azurol S (CAS) assay (**B**)[13]. Each treatment was performed in triplicates, and data expressed as mean ± standard deviation. Different lowercase letters above columns indicate significant differences among treatments at *P* ≤ 0.05 based on Tukey’s HSD test. “Ac, Fl and Ba” represent *Achromobacter* sp. NAU112411, *Flavobacterium* sp. NAU112421, and *Bacillus* sp. NAU1124221, respectively.

**Reference**

[1]	X. Sun, Z. Xu, J. Xie, V. Hesselberg-Thomsen, T. Tan, D. Zheng, M. L. Strube, A. Dragoš, Q. Shen, R. Zhang, *ISME J.* **2022**, *16* (3), 774.

[2]	X. Ye, Z. Li, X. Luo, W. Wang, Y. Li, R. Li, B. Zhang, Y. Qiao, J. Zhou, J. Fan, *Microbiome* **2020**, *8*, 1.

[3]	Z. Li, X. Ye, P. Chen, K. Ji, J. Zhou, F. Wang, W. Dong, Y. Huang, Z. Zhang, Z. Cui, *Biol. Control* **2017**, *110*, 10.

[4]	a) C. N. Tango, S. Akkermans, M. S. Hussain, I. Khan, J. Van Impe, Y.-G. Jin, D. H. Oh, *Food Microbiol.* **2018**, *76*, 287; b) D. Ren, J. S. Madsen, S. J. Sørensen, M. Burmølle, *ISME J.* **2015**, *9* (1), 81; c) D. Ren, J. S. Madsen, C. I. de la Cruz-Perera, L. Bergmark, S. J. Sørensen, M. Burmølle, *Microb. Ecol.* **2014**, *68* (1), 146.

[5]	S. Agarwal, D. W. Hunnicutt, M. J. McBride, *P. Natl. Acad. Sci.* **1997**, *94* (22), 12139.

[6]	a) B. Alvarez, P. Secades, M. McBride, J. Guijarro, *Appl. Environ. Microb.* **2004**, *70* (1), 581; b) Y. Shi, C. Li, H. Yang, T. Zhang, Y. Gao, M. Chu, J. Zeng, Q. Lin, OuTiKuEr, Y. Li, *J. Microbiol.* **2017**, *55* (4), 267.

[7]	J. N. Wilking, V. Zaburdaev, M. De Volder, R. Losick, M. P. Brenner, D. A. Weitz, *P. Natl. Acad. Sci.* **2013**, *110* (3), 848.

[8]	M. K. Swamy, M. S. Akhtar, U. R. Sinniah, *Root Exudates and Their Molecular Interactions with Rhizospheric Microbes*, **2016**.

[9]	N. Zhang, D. Wang, Y. Liu, S. Li, Q. Shen, R. Zhang, *Plant Soil* **2014**, *374* (1-2), 689.

[10]	V. J. Carrión, J. Perez-Jaramillo, V. Cordovez, V. Tracanna, M. de Hollander, D. Ruiz-Buck, L. W. Mendes, W. F. J. van Ijcken, R. Gomez-Exposito, S. S. Elsayed, P. Mohanraju, A. Arifah, J. van der Oost, J. N. Paulson, R. Mendes, G. P. van Wezel, M. H. Medema, J. M. Raaijmakers, *Science* **2019**, *366* (6465), 606.

[11]	J. Sasse, E. Martinoia, T. Northen, *Trends Plant Sci.* **2018**, *23* (1), 25.

[12]	F. Ahmad, I. Ahmad, M. Khan, *Microbiol. Res.* **2008**, *163* (2), 173.

[13]	B. C. Louden, D. Haarmann, A. M. Lynne, *J Microbiol. Biol. Edu.* **2011**, *12* (1), 51.
